# De Novo Detection of Clonal Structure and Evolution in Single-Cell and Spatial Transcriptomes

**DOI:** 10.3390/ijms262311428

**Published:** 2025-11-26

**Authors:** Shihao Bai, Xianbin Su, Ziyao Chen, Ze-Guang Han

**Affiliations:** Key Laboratory of Systems Biomedicine (Ministry of Education), Shanghai Center for Systems Biomedicine, Shanghai Jiao Tong University, Shanghai 200240, China; b369259046@sjtu.edu.cn (S.B.); unicornczy@sjtu.edu.cn (Z.C.)

**Keywords:** clone inference, single-cell mutation, single-cell transcriptomes, spatial transcriptomes, mutational signature

## Abstract

Tumors are composed of cellular populations with distinct genotypes and phenotypes, which dynamically evolve over time and during treatment. This process is known as clonal evolution, and it is difficult to reveal fine-scale clonal structure with traditional bulk sequencing. Although single-cell genome sequencing could enable reconstruction of tumor clonal evolution, it remains technically challenging and the number of single cells profiled is generally insufficient due to high cost. To address this issue, we developed scClone, a computational toolkit that integrates variant detection and genotype inference for single-cell RNA-seq (scRNA-seq) and spatial transcriptomic data. It further provides interactive visualization of clonal structure and dynamic evolution. scClone addresses key limitations inherent to scRNA-seq, such as expression drop-out and allelic imbalance, and incorporates cell type or state annotation with mutational signature analysis to enable comprehensive profiling of tumor heterogeneity. scClone demonstrated robust performance across multiple datasets—generated from both full-length and fragmented RNA sequencing—by accurately reproducing mutation profiles and resolving clonal mixtures in myeloma, hepatocellular carcinoma and pancreatic cancer. Additionally, scClone has been applied to spatial transcriptomics, enabling the delineation of clonal structures within histological sections from ovarian cancer and cutaneous squamous cell carcinoma. In summary, our results demonstrate that scClone can extract genetic information from scRNA-seq datasets, thereby successfully establishing genotype–phenotype associations at the single-cell level and providing insights into the clonal evolution of tumors.

## 1. Introduction

The progression of cancer is closely related to Darwinian evolution. Genetic or epigenetic variations could alter the molecular phenotypes of individual cells. Tumors at the time of diagnosis typically consist of multiple cell populations with distinct genetic profiles, which are referred to as clones [1]. Clonal evolution describes the process through which tumor clones undergo phylogenetic diversification, driven by selective pressures exerted by the tumor microenvironment (TME) or therapeutic interventions, ultimately shaping the tumor’s evolutionary trajectory. Clonal diversity within tumors drives tumor heterogeneity, leading to differential growth advantages, metastatic potential, therapeutic responsiveness, and prognostic outcomes, which collectively underlie clinical challenges in cancer management. Thus, precise delineation of the clonal origin and architectural dynamics is critical for reconstructing tumor evolutionary trajectories and elucidating their clinical implications in therapeutic resistance, metastatic dissemination, and disease recurrence [2].

Methods such as fluorescence in situ hybridization (FISH) and immunohistochemistry (IHC) allow us to vaguely observe the clonal structure of tumors. In addition, techniques such as CRISPR-based lineage tracing and fluorescent labeling methods [3], which track cell division and proliferation during tumor progression, are striving to reveal the process of tumor clonal evolution. The advent of high-throughput DNA sequencing has enabled the application of bioinformatics pipelines to deconvolve hidden evolutionary trajectories from bulk genomic sequencing data, primarily by inferring clonal architectures through variant allele frequency (VAF)-based clustering and phylogenetic reconstruction. However, current strategies for bulk-level clonal evolution analysis suffer from an inherent flaw: they use the clustering of mutations to infer tumor clones, but the essence of tumor clones is the clustering of cell lineages [4]. For example, a subclone is defined as a genetically distinct group of tumor cells resulting from a clonal expansion; however, in practice, it is often detected by a group of mutations whose variant allele frequency (VAF) equals approximately 0.25 in bulk sequencing. Inferring the complex clonal structure within tumors using one-dimensional bulk VAF values inherently introduces inaccuracies and deviates from the true results, as the nonlinear relationship between VAFs and clonal propagation dynamics (e.g., clonal sweeps, neutral drift, or selective sweeps) could lead to either false negatives (underdetection of subclones) or false positives (overestimation of clonal dominance). This suggests that clonal analysis indeed requires higher-resolution investigation.

Single-cell multi-omics provides unprecedented resolution to dissect the mechanisms underlying occurrence and progression of tumors, revealing clonal evolution trajectories, phenotypic plasticity, and cell-state transitions that are obscured in bulk analyses. Single-cell DNA sequencing (scDNA-seq) is the most direct method for revealing tumor clonal architectures at the cellular level, but its widespread adoption is constrained by technical bottlenecks, including ultralow DNA input per cell, amplification-induced artifacts, and high cost per cell [5]. To circumvent these issues, some suboptimal methods have been developed, such as isolating clonal cell populations from tissues or in vitro culture, followed by bulk whole-genome/exome sequencing (WGS/WES) [4,6,7]. However, these methods retain the fundamental flaws of bulk sequencing, thereby compromising their utility in deconvolving tumor heterogeneity. Single-cell-targeted sequencing offers another cost-effective approach, but its utility is limited by the number of detected cells and genetic loci [8,9], as well as dependency on bulk sequencing-derived prior knowledge for panel design. In addition, the technologies mentioned above lack the capacity for simultaneous interrogation of cancer cell genotypes and phenotypes [10].

Although techniques for sequencing both DNA and RNA from the exact same cell have been developed, this strategy remains technically challenging in human samples [11,12,13,14]. Some methods have been devised to infer the relationship between scDNA-seq and single-cell RNA sequencing (scRNA-seq), but still on different cells [15,16]. One low-resolution method for linking cell genotype and phenotype involves predicting DNA copy number variations (CNVs) based on scRNA-seq data, followed by hierarchical clustering of the CNVs to infer clonal evolution [17,18,19]. However, this method implicitly assumes a linear evolutionary model, where branch points in the resulting dendrogram are interpreted as chronological events, thereby overlooking the confounding effects of convergent evolution, copy number neutral losses of heterozygosity (LOH), and selective pressures that may distort the relationship between CNV distance and temporal acquisition. Although the distance dendrograms in such analyses resemble phylogenetic trees, they actually fail to reflect clonal evolution [20].

It is technically feasible to directly detect somatic mutations in RNA sequencing reads from scRNA-seq data, which enables cell lineage tracing through transcriptional characteristics with annotation information. However, mutation detection and clonal inference from scRNA-seq data are limited by many factors such as differential expression, allelic imbalance, RNA editing, limited sequencing coverage or depth, sequencing artifacts, etc. [21]. It should be noted that, although RNA editing has been reported as an important phenotypic feature of cells that may be used for subclone identification [22,23], large numbers of RNA editing events may mask sparse genomic evidence in scRNA-seq data.

**Figure 1:**
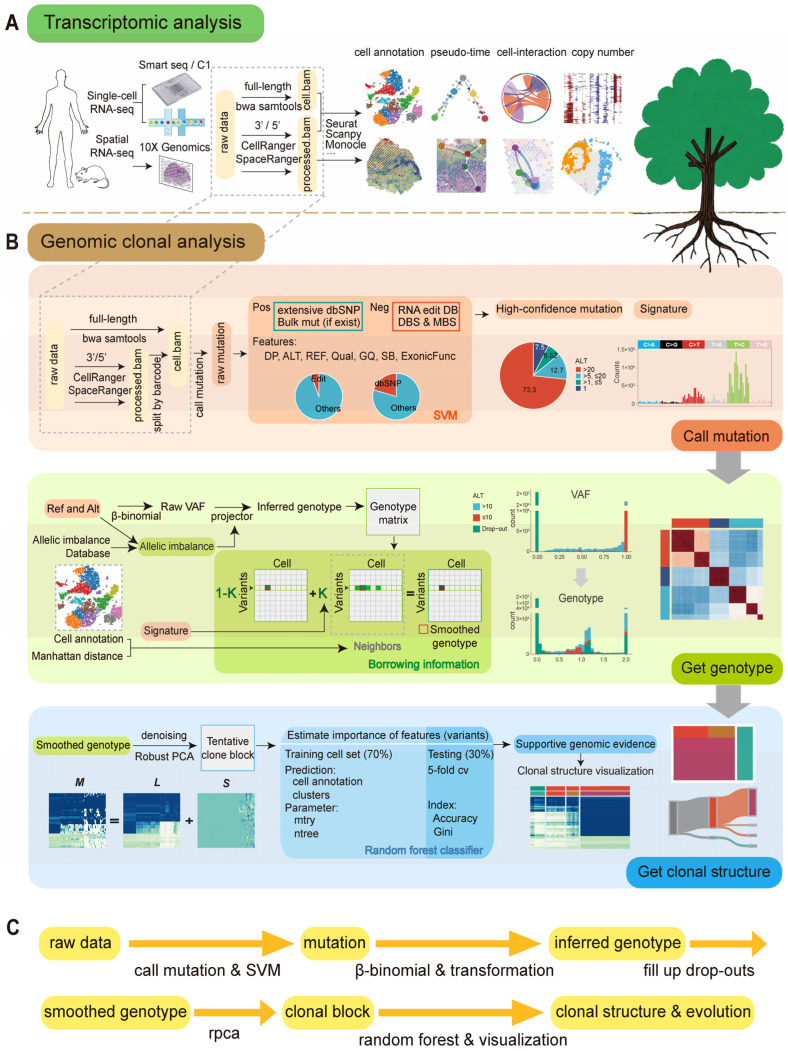
Workflow of scClone and its relationship with single-cell transcriptome analysis. (**A**) Schematic diagram of single-cell and spatial transcriptome sequencing processes and bioinformatics analysis methods. (**B**) Detailed workflow of scClone. SVM (support vector machine); DBS (doublet base substitutions); MBS (multi-base substitution); VAF (variant allele frequency); Qual (phred-scaled quality score); GQ (genotype quality); GQX (empirically calibrated genotype quality score for variant sites); ExonicFunc (exonic function); M, L, and S represent the optimized matrix, the low-rank matrix, and the sparse matrix, respectively. (**C**) Simplified workflow of scClone.

To address these challenges, we developed a full-process computational toolkit, scClone, for mutation detection and clonal evolution analysis based on single-cell transcriptome data. scClone will directly process raw sequencing reads to detect somatic mutations, impute drop-outs, and visualize clonal structures and evolutionary relationships. Our toolkit is not limited by methods of RNA library preparation or sequencing platforms, and it effectively takes advantage of single-cell transcriptomic annotations and bulk sequencing-derived mutational signatures. Currently, single-cell transcriptome analysis has matured significantly, supported by a vast volume of data, and now resembles a sprawling tree with intricate branches and lush foliage (Figure 1A). Clonal evolution, a cornerstone of cancer research, mirrors the intricate and interwoven root system of a thriving tree, where hidden branches fuel the tree’s resilience and expansion. Our approach will add a new dimension for deconvolution of clonal architecture with single-cell transcriptomic profiling, provide new insights into tumor evolution, and hopefully contribute to future clinical applications.

## 2. Results

### 2.1. Benchmark: Assessing the Effectiveness and Usability of scClone

To assess the efficiency of our toolkit, we initially used single-cell transcriptomic data of myeloma cells obtained via C1 platform as a benchmark [24]. The dataset included eight samples from six patients, including bone marrow, additional extramedullary (EM) ascites and pleural effusion samples. All cells were sorted using CD138-based flow cytometry and data were derived from the same batch. Here we discarded cell annotations and processed all myeloma cells in parallel to infer the clonal structure. Given that these myeloma cells experienced different selective pressures across patients and tissues, eight samples served as a good reference for the clonal structure inference to assess our toolkit. We also selected another 10× Genomics dataset from a cutaneous squamous cell carcinoma (cSCC) patient, and its availability of bulk whole-exome sequencing (WES) data from paired tumor and adjacent normal tissues in the same patient provided further information for method assessment [25].

**Figure 2:**
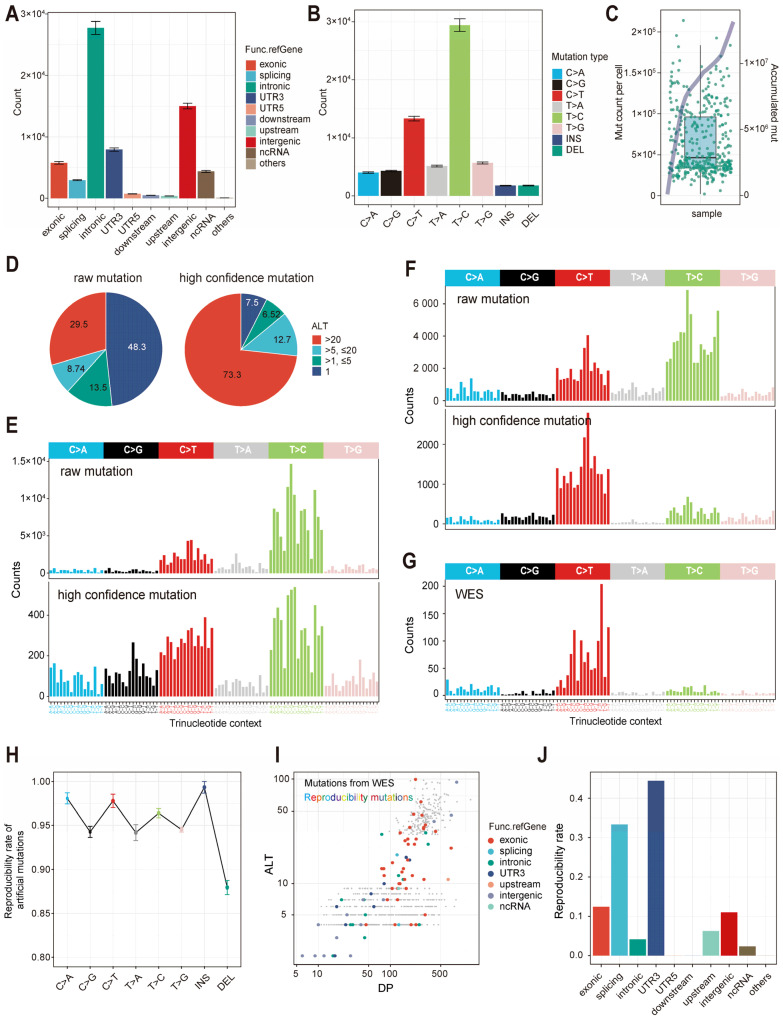
Detection of mutations in the transcriptome by scClone. (**A**) Statistics of functional annotation for raw mutations in myeloma cells detected by scClone. Error bars represent standard errors. (**B**) Statistics of the nucleotide substitution types for raw mutations in myeloma cells. Error bars represent standard errors. (**C**) Number of mutations in myeloma cells. Box and scatter plots show the number of mutations in each myeloma single cell (*y*-axis on the left), and the line shows the cumulative number of mutations in all myeloma cells (*y*-axis on the right). (**D**) Fractions of mutations with ALT reads before and after SVM filtering in myeloma dataset, with raw mutations on the left and high-confidence mutations on the right. (**E**) Mutational signatures in 96 trinucleotide contexts before and after SVM filtering in myeloma cells. (**F**) Mutational signatures in the cutaneous squamous cell carcinoma (cSCC) dataset before and after SVM filtering. (**G**) Mutational signatures in cSCC via bulk-level exome sequencing. (**H**) Reproducibility of artificially introduced mutations in myeloma cells. The standard deviation from an independent repeated experiment has been labeled. (**I**) Mutations identified by WES in cSCC. Colored dots indicate mutations detected by scClone. (**J**) Reproducibility of scClone-detected mutations in cSCC relative to the WES.

Regardless of the scRNA-seq library preparation chemistries, the majority of the raw mutations were located in intronic, intergenic and UTR3 regions, followed by exonic regions (Figure 2A and Figure S1A). This mutation distribution pattern was generally consistent with traditional genomic mutational profiling, with the larger genomic fractions of intronic and intergenic calls arising from incompletely spliced pre-mRNA, single-cell 3’/5’UTR capture and amplification artifacts. Additionally, both datasets exhibited a high prevalence of T > C and C > T mutations (Figure 2B and Figure S1B). Although previous studies have shown that clock-like and DNA damage signatures can also lead to C > T and T > C mutations, the substantial number of mutations observed here suggests that a considerable proportion of these mutations are likely attributable to RNA-editing events [22,26,27]. The full-length transcriptome data from the C1 platform exhibited an average of ~50,000 mutations per cell (Figure 2C), whereas the 10 × 3′ sequencing data showed only ~3000 mutations per cell (Figure S1C). Upon analyzing the cumulative mutation counts across all single cells, we observed a near-linear relationship between total mutation count and cell number, which suggested that mutations were rarely shared among cells (Figure 2C and Figure S1C).

After processing with SVM, the proportion of mutation sites with read depth > 20 increased from 29.5% to 73.3%, whereas the proportion of mutations with a depth of 1 decreased from 48.3% to 7.5% (Figure 2D). These retained low-depth mutations might subsequently be utilized to infer the genotypes of drop-outs. The filtering also led to a notable reduction in T > C and C > T substitutions, which were primarily attributed to RNA editing (Figure 2E). As the cSCC samples had available WES data, it was observed that the mutational signature obtained after the SVM filtering more closely resembled the signatures derived from the bulk data, with cosine similarity raised from 0.47 to 0.79, which indirectly reflected the reliability of scClone in calling mutations and the usability of transcriptional mutation signatures (Figure 2F,G). To assess the usability of scClone in mutation detection, we artificially introduced 1000 mutations into randomly selected myeloma cells and performed six independent replicates (Supplementary Materials). Across all categories of mutations, scClone consistently achieved reproducibility rates exceeding 85% (Figure 2H). For the cSCC, we evaluated mutation reproducibility directly against bulk WES data; among the 880 mutations, 78 (8.9%) were detected in the scRNA-seq data processed by scClone (Figure 2I,J). Given the extremely low coverage of the 3′/5′ sequencing regions, this level of concordance confirmed the adequacy of scClone for mutation detection. However, the limited mutations of cSCC epithelial cells in 3′ sequencing data precluded further analysis. We further evaluated the latest scRNA-seq somatic mutation detection tool SComatic in the cSCC dataset and all mutations from SComatic were retained within the scClone mutation list. However, the limited yield rendered these calls insufficient for robust downstream clonal-evolution inference (Figure S2A–H).

**Figure 3:**
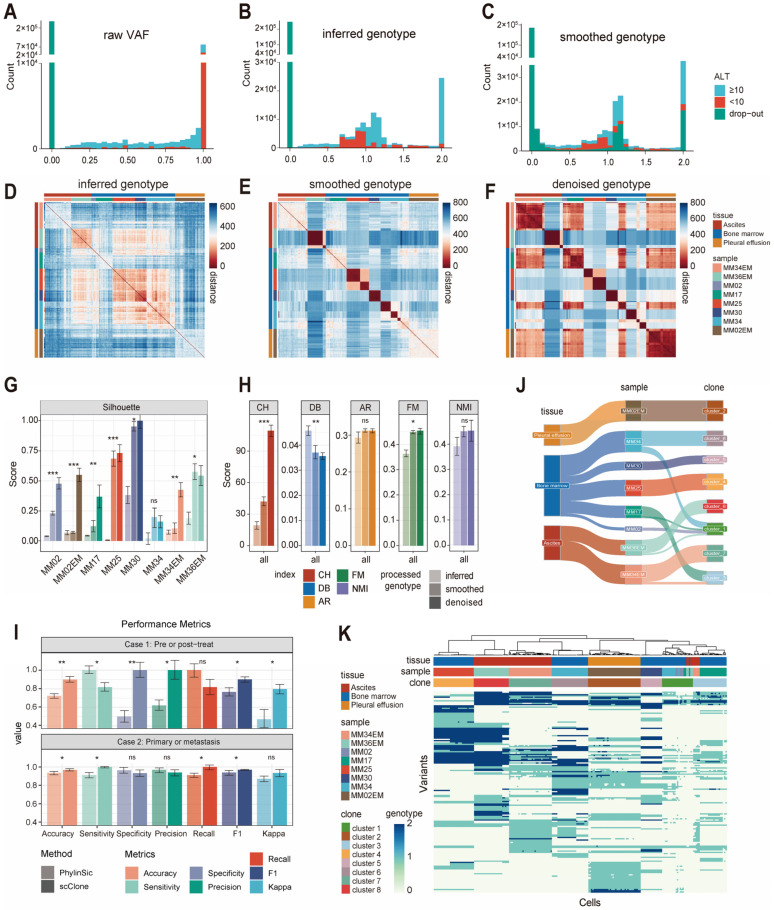
Evaluation of scClone performance in the myeloma dataset. (**A**) Raw VAF of high-confidence mutation sites. VAF ranges from 0 to 1. (**B**) Inferred genotypes. Genotype 0: wild-type; 1: heterozygous mutation; 2: homozygous mutation. (**C**) Smoothed genotypes after filling in drop-outs. (**D**–**F**) Heatmap of Manhattan distances between cell genotypes after different processing steps: inferred genotypes (**D**), smoothed genotypes (**E**), and denoised genotypes (**F**). (**G**) Silhouette scores for different samples, with most samples showing improved scores after each of the three processing steps for genotypes. (**H**) Evaluation of genotype clustering similarity to sample clustering using multiple metrics during the stepwise analysis. AR: Adjusted Rand; CH: Calinski–Harabasz; DB: Davies–Bouldin; FM: Fowlkes–Mallows; NMI: Normalized Mutual Information. (**I**) Benchmarking scClone against PhylinSic for clonal identification in pre- vs. post-treatment and primary vs. metastatic tumors. (**J**) Sankey diagram showing the relationship between tissue origin, sample identity and clonal clusters from scClone. (**K**) Visualization of scClone output, with each row representing a mutation and each column representing a cell. Heatmap colors indicate genotypes. The standard deviation from an independent repeated experiment has been labeled. ANOVA (analysis of variance) test results are shown in (**G**,**H**). Student’s *t*-test results are shown in (**I**) (ns: *p* > 0.05; *: *p* ≤ 0.05; **: *p* ≤ 0.01; ***: *p* ≤ 0.001).

In analysis of myeloma samples, the inferred genotypes exhibited obvious heterozygous peaks compared with raw VAF values (Figure 3A,B). The Manhattan distances of these inferred genotypes revealed indistinct clusters among cells from different sample origins (Figure 3D). Interestingly, after erasing cell annotation and filling in drop-outs based on the computed distances, the heatmap depicting the processed genotypes became more compact, with more pronounced differences among samples (Figure 3C–F). These outcomes could be attributed to the reliability of mutations, the rational inference of genotypes, and the effectiveness of the Manhattan distance in reflecting cellular clonal relationships. We assessed the clustering performance based on the progressively refined genotypes and found that the silhouette score improved in almost all samples (Figure 3G). Additionally, we evaluated the concordance between clusters and samples using various scores (CH, DB, AR, FM, and NMI), which indicated that the progressively refined processes increasingly approximated the true clone structure (Figure 3H). We also compared the impact of different thresholds for defining neighboring cells on clustering performance and found that scClone was not sensitive to these thresholds (Figure S3).

Next, we compared scClone with PhylinSic [20], another toolkit that also infers clonal evolution de novo from single-cell transcriptomes. In both datasets—pre- vs. post-treatment myeloma and primary vs. metastatic myeloma—scClone achieved overall superior clonal identification, accurately detecting clonal lesions from the same patient across different time and locations, and delivered better accuracy, recall, and F1-score (*p* < 0.05) (Figure 3I). We further assessed the clonal identities of single cells and determined the optimal hyperparameters to generate a low-complexity clonal structure within the random forest framework (Figure S4A–F). Five-fold cross-validation indicated the probability that cells from different samples were correctly classified (Figure S4D).

Ultimately, eight mutation-based tumor clonal clusters were formed (Figure 3J,K). Utilizing sample origins as independent clonal labels for orthogonal validation, we found that solely relying on scClone from raw data to final clonal identification without any sample information, six out of the eight genomic clonal clusters (cluster 2, 4, 5, 6, 7, and 8) accurately reflected their respective sample origins. Specifically, cells from different tissues of the same patient were clearly distinguished: the bone marrow (MM02) and pleural effusion samples (MM02EM) from patient MM02 were assigned to different clusters. The same situation was observed in the two samples from patient MM34. However, clonal clusters 1 and 3 were composed of cells from multiple sample origins. This phenomenon might necessitate the incorporation of additional genomic evidence or cell numbers to enhance the robustness of scClone and thereby improve the accuracy and resolution of clonal analysis.

### 2.2. Identification of Subclones Among Single Cells by scClone

**Figure 4:**
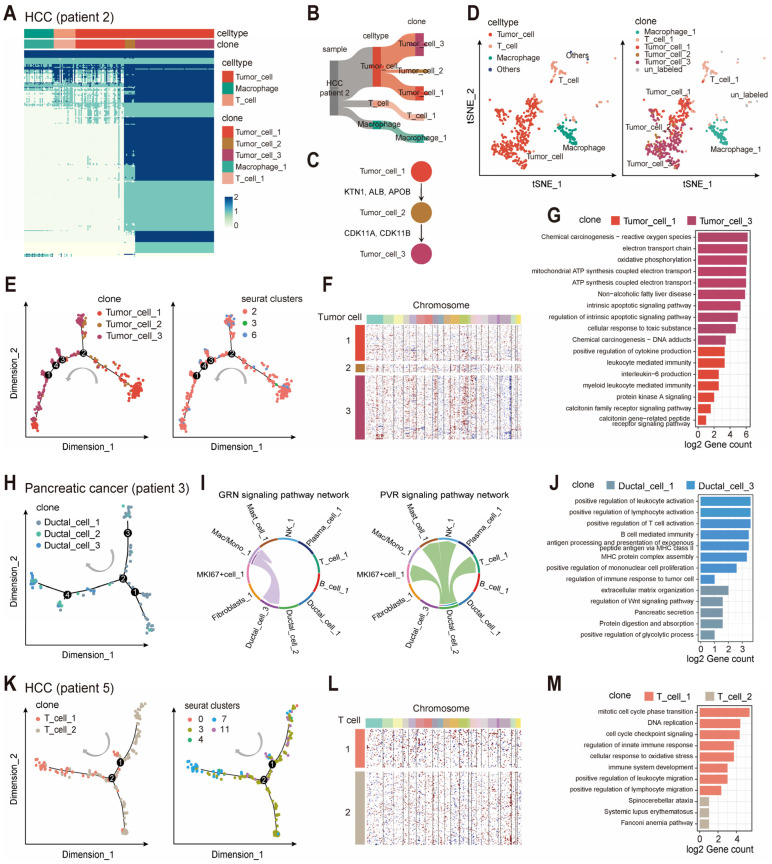
Clonal clusters identified via scClone have functional differences. (**A**) “Variant-cell” map of an HCC patient (patient 2) based on scClone. (**B**) Sankey diagram showing cell types and clonal clusters in HCC patient 2. (**C**) Schematic of tumor clonal progression in HCC patient 2 with non-synonymous key mutations. (**D**) tSNE map of HCC patient 2, with cell type (left) and projected clonal cluster information (right). (**E**) Pseudo-time trajectory of tumor cells in HCC patient 2, with clonal cluster (left) and transcriptional cluster information (right). (**F**) Whole-genome copy number variations (CNV) inferred from a single-cell transcriptome for tumor cells in HCC patient 2, with each row representing a cell and each column representing a chromosomal bin. (**G**) Enrichment analysis of differentially expressed genes between tumor clones in HCC patient 2. (**H**) Pseudo-time trajectory of ductal cells in a pancreatic cancer patient (patient 3), with clonal cluster information. (**I**) Interaction of signaling pathways among different cell clones in pancreatic cancer patient 3. (**J**) Enrichment analysis of differentially expressed genes between clonal clusters in pancreatic cancer patient 3. (**K**) Pseudo-time trajectory of T cells in HCC patient (patient 5), with clonal cluster (left) and transcriptional cluster information (right). (**L**) Whole-genome CNV inferred from single-cell transcriptome for T cells in HCC patient 5. (**M**) Enrichment analysis of differentially expressed genes between clonal clusters of T cells in HCC patient 5.

Aiming to assess the ability of scClone in detecting clonal structures in tumor cells, we applied scClone to full-length single-cell transcriptomic data of 800 cells from a hepatocellular carcinoma patient (HCC patient 2) [8]. According to the heatmap generated by scClone, three genomic mutational clusters were identified within the tumor cell population, clearly showing a stepwise accumulation of mutations (Figure 4A,B). Tumor cell cluster 1 tended to represent an early subclone with the lowest degree of genetic variation among tumor cells, and exhibited genotypes more closely resembling those of immune cells such as macrophages and T cells. In contrast, tumor cell cluster 3 exhibited the highest mutation count and represented the most recently evolved subclone, which may have acquired a selective advantage and subsequently expanded. Tumor cell cluster 2 occupied an intermediate stage in the evolutionary trajectory from tumor cell cluster 1 to tumor cell cluster 3. By examining the mutated genes, we found that tumor cells progressively acquired non-synonymous mutations in genes such as KTN1, ALB, APOB, CDK11A, and CDK11B (Figure 4C). We then analyzed single-cell target sequencing (scTarget-seq) data from the HCC patient 2 [8]: 50 cells and 55 variant positions yielded a cell-by-mutation matrix that resolved two tumor subclones whose composition resembled the tumor cell clusters 1 and 3 identified by scClone (Figure S5). Given the limited throughput of scTarget-seq, the intermediate tumor cell 2 was not captured. Despite the scRNA-seq and scTarget-seq sampling different tumor sectors, the convergent clonal picture corroborates the robustness of the scClone reconstruction.

We then mapped the clonal information derived from scClone onto a two-dimensional tSNE map, where the distances between cells reflected transcriptomic similarities (Figure 4D). Notably, tumor cell clusters 1 and 3 were mapped to two distinct transcriptomic clusters on the tSNE map, while tumor cell cluster 2 demonstrated overlap with both clusters. Interestingly, tumor cell cluster 2 represented an intermediate state in both clonal evolution and transcriptomic profiles. This observation suggested a close relationship among the three clonal clusters with different genetic variations and the transcriptional expression profiles. Indeed, it was reasonable to observe distinct expression profiles among tumor subclones, given that subclone-specific mutations might exert varying functional impacts on the transcriptomic landscape.

We further investigated the relationship between scClone-derived clonal clusters and multiple concepts derived for single-cell transcriptomes. Mapping clonal cluster information onto a branched trajectory of pseudo-time analysis, we observed that tumor cell cluster 3 was concentrated on the left branches, while tumor cell cluster 1 and 2 did not show significant enrichment, suggesting a lack of consistency between mutation-based clonal clusters and pseudo-time trajectories (Figure 4E). One explanation was that pseudo-time analysis like Monocle2 identified continuous gene expression patterns to infer “time,” which might not be on the same timescale compared with mutation accumulation. Thus, the two approaches provided complementary information for better understanding of “time” and “evolution” at multiple levels. We further calculated CNVs from the gene expression profiles by inferCNV [28], and the results were partially aligned with scClone clonal clusters. Tumor cell cluster 3 exhibited widespread chromosome 19 deletions and frequent amplifications on chromosomes 8 and 12, while no significant CNVs were detected in tumor cell cluster 1 and 2 (Figure 4F). As clonal clusters from scClone were based on genomic mutations, the resolution was relatively higher than CNV clusters from transcriptional profiles.

From enrichment analysis of clonal cluster-specific expressed genes, we found functional differences in metabolism between tumor cell clusters 1 and 3. Tumor cell cluster 1 showed enrichment with immunity and cell signaling, involving leukocyte-mediated immunity and production of cytokines and interleukin-6 (Figure 4G). These immune-related signatures in cluster 1 may have important clinical implications—interleukin-6 is a well-established pro-inflammatory cytokine associated with aggressive tumor phenotypes and poor prognosis in hepatocellular carcinoma (HCC), suggesting that tumor cell cluster 1 with higher infiltration or activity may carry an increased risk of recurrence or distant metastasis [29,30]. In contrast, tumor cell cluster 3 focused more on energy metabolism, cellular stress and apoptosis, as well as several disease-related pathways like non-alcoholic fatty liver disease and chemical carcinogenesis. Tumor cell cluster 3 highlights a potential link to metabolic-associated HCC subtypes, which are increasingly prevalent clinically and require tailored therapeutic strategies; meanwhile, the emphasis on cellular stress and apoptosis may imply cluster 3 cells are more vulnerable to metabolic-targeted agents or oxidative stress-inducing therapies, providing a direction for precision treatment [31]. By investigating cell–cell interactions by CellChat [32], we found that tumor cell 3 had more MHC-I signaling interactions with tumor cell cluster 1 and macrophages, and tumor cell cluster 2 had PECAM1 signaling interactions with macrophages and exhibited a strong WNT signaling pathway interaction with other cell clusters (Figure S6A,B). Enhanced MHC-I-mediated crosstalk suggests immunotherapy responsiveness, as MHC-I supports antigen presentation and T cell-activation serves as a predictive biomarker for immune checkpoint inhibitor patient selection. Additionally, cluster 2’s prominent WNT signaling implies a role in tumor progression and stemness, closely linked to HCC recurrence and poor survival [33]. Collectively, these cluster-specific features reveal HCC’s molecular heterogeneity and offer actionable insights for clinical stratification, prognostic assessment, and personalized therapy.

We further applied scClone to pancreatic cancer scRNA-seq data from the 10× Genomics platform (patient 3), which contained more cells and retained greater transcriptomic diversity, including ductal cells, fibroblasts and multiple types of immune cells [34] (Figure S7A–D). However, due to the lower amount of mRNA captured per cell in the 10× 5′ chemistry and the requirement of sufficient mutations for clonal identification, a higher proportion of single cells were filtered out by scClone compared to full-length data. Ductal cells were divided into three clonal clusters by scClone, with a gradual accumulation of mutations across these clusters (Figure S7A). This dynamic process was also evident in pseudo-time trajectory analysis, where ductal cell cluster 1 was enriched in the early stages and ductal cell cluster 3 at the terminal end (Figure 4H). We also uncovered different transcriptional activity levels of the GRN and PVR signaling pathways among the three clonal clusters (Figure 4I and Figure S7E). Ductal cell cluster 1 was primarily enriched with metabolic pathways, whereas ductal cell cluster 3 exhibited substantial activity related to immunity and antigen presentation, suggesting ductal cells derived from cluster 3 could be more responsive to immunotherapies, providing a potential marker for treatment selection (Figure 4J).

### 2.3. scClone Describes the Evolutionary Trajectories of Immune Cells

The concept of clones in immune cells, particularly T cells and B cells, is traditionally defined based on TCR and BCR clones, which are determined by the rearrangement of the VDJ regions in the genome. These clones are used to describe the evolution of T cells and B cells to recognize specific antigens. In fact, any proliferating cells, whether tumor cells or non-tumor cells, will inevitably accumulate mutations during the DNA replication process. In a full-length single-cell transcriptomic dataset from another HCC patient (patient 5), scClone identified two clusters of T cells (Figure S8A–D). These two T-cell clusters exhibited transcriptomic differences in pseudo-time analysis (Figure 4K), and their differences were also supported by copy number inference, with T-cell cluster 2 harboring genomic amplification at 19q regions (Figure 4L). For enrichment analysis of genes differentially expressed between the two clusters, T-cell cluster 1 was primarily involved in cell proliferation and immune activity, indicating its participation in tumor immune infiltration, while T-cell cluster 2 was associated with limited pathways (Figure 4M). Additionally, T-cell cluster 1 exhibited more interactions with other cells through the CD96 signaling pathway and communicated with tumor cells via EPGN (Figure S8E). Notably, CD96 signaling dysregulation in T cells has been linked to impaired anti-tumor immunity and poor clinical outcomes, implying that targeting this pathway could enhance the therapeutic effect for this patient, providing a personalized treatment direction [35,36].

### 2.4. scClone Enables Clonal Cluster Identification in Spatial Transcriptomics

The high similarity between single-cell and spatial transcriptomics data allows easy application of scClone to spatial transcriptomics data. The key difference between the two approaches lies in the resolution, as spatial transcriptomics are based on ROI (region of interest) spots, each containing multiple cells. This makes it difficult to precisely define the cell types within a spot. The requirement for enough mutations in a spot for clonal inference inevitably leads to the filtering out of some spots, resulting in the loss of information at these sites, which cannot be recovered. These two factors make clonal inference in spatial transcriptomics more challenging and lower in resolution.

**Figure 5:**
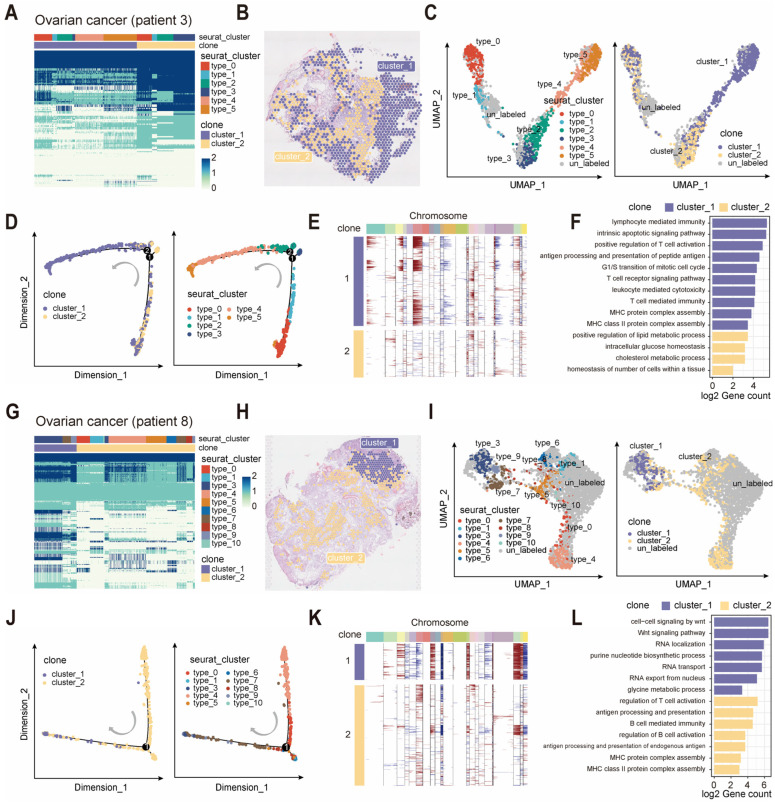
scClone enables clonal cluster identification in spatial transcriptomics. (**A**) “Variant-cell” map of ovarian cancer patient (patient 3) based on spatial transcriptome from scClone. (**B**) Clonal structure of ROI spots displayed on pathological sections from patient 3. (**C**) UMAP of single cells from patient 3, with cell type (left) and clonal cluster information (right). (**D**) Pseudo-time trajectory of ROI spots in patient 3, with clonal cluster (left) and transcriptional cluster information (right). (**E**) Whole-genome CNV inferred from single-cell transcriptome for patient 3, with each row representing a spot and each column representing a chromosomal bin. (**F**) Enrichment analysis of differentially expressed genes between clonal clusters in patient 3. (**G**) “Variant-cell” map of ovarian cancer patient (patient 8). (**H**) Clonal structure of ROI spots displayed on pathological sections from patient 8. (**I**) UMAP of single cells from patient 8, with cell type (left) and clonal cluster information (right). (**J**) Pseudo-time trajectory of ROI spots in patient 8, with clonal cluster (left) and transcriptional cluster information (right). (**K**) Whole-genome CNV inferred from single-cell transcriptome for patient 8. (**L**) Enrichment analysis of differentially expressed genes between clonal clusters in patient 8.

We first demonstrated the ability of scClone to identify clonal clusters in the ovarian cancer spatial atlas. For two cases of ovarian cancer spatial transcriptomics data from patients 3 and 8 [37], scClone identified two subclones in each patient that showed distinct clustering on pathological sections (Figure 5A,G). In patient 3, two clonal clusters were consistent with the histological sections, suggesting a difference in tumor morphology for the two subclones (Figure 5B). UMAP results of the expression profiles revealed the differential expression between the two subclones in patient 3 (Figure 5C), together with significantly different enrichment along the pseudo-time trajectory (Figure 5D). Cluster 1 tended to exhibit higher tumor purity and malignancy as evidenced by more amplifications of chromosome 6 in copy number inference, while cluster 2 appeared relatively normal (Figure 5E). Cluster 1 involved the activation of immune cells, antigen processing, apoptosis and cell cycle regulation, while cluster 2 was associated with metabolism and homeostasis (Figure 5F). This evidence indicated that tumor clone regions derived from cluster 1 have faster tumor progression and a higher antigen response.

In ovarian cancer patient 8, we similarly identified that cluster 1 had more accumulated mutations and a relatively normal cluster 2 (Figure 5G). Cluster 1 was prominently clustered in the upper region of the histological section, while cluster 2 spanned across a much larger region (Figure 5H). Both clonal clusters showed significantly different expression and pseudo-time enrichment (Figure 5I,J). In addition to differences in mutations revealed by scClone, these two subclones displayed clear distinctions in copy number inference, with cluster 1 exhibiting amplifications of chromosomes 3, 8, 13, 20 and 21, along with deletions of chromosomes 10 and 22 (Figure 5K). Cluster 1 was characterized by RNA transport and modification, nucleotide metabolism and intercellular signaling pathways such as the Wnt signaling pathway. Cluster 2 was mainly enriched with antigen processing and presentation, and immune activation (Figure 5L). Thus, the clonal structure identified in spatial transcriptomics by scClone was generally consistent with the histological sections and provided special biological insight.

### 2.5. Integration of scClone and Transcriptomic Information Reveals High-Resolution Clonal Structures

**Figure 6:**
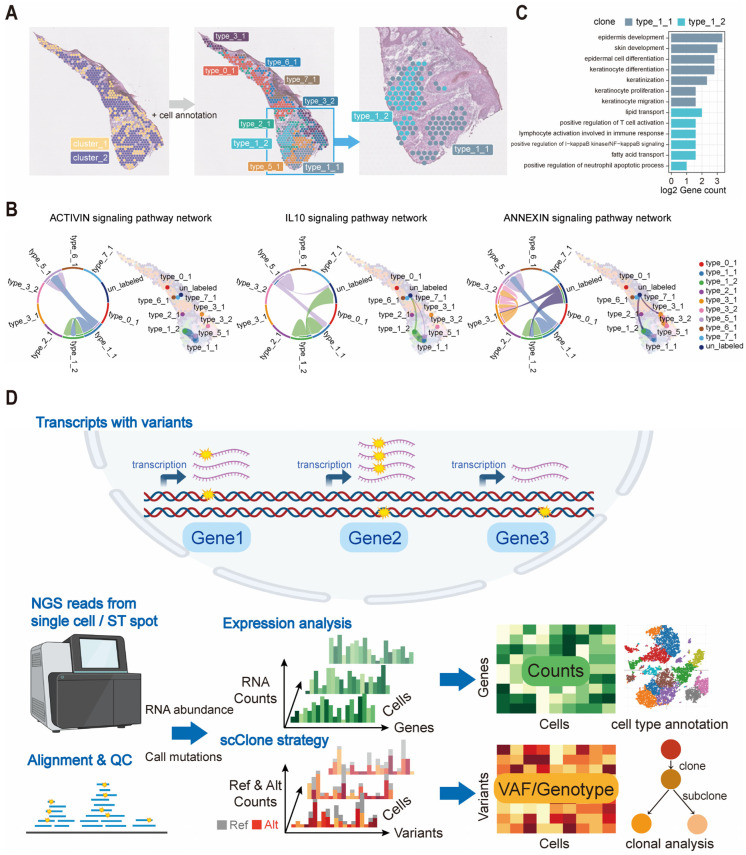
Functional interpretation of scClone-derived clonal clusters in spatial transcriptomics. (**A**) Spatial clonal structure of cSCC (patient 4) (left), clonal structure combined with transcriptomic clusters (middle), and zoomed-in view of the two clonal clusters of type_1 (right). (**B**) Interaction of signaling pathways between spatial transcriptomic spot clones in patient 4. (**C**) Enrichment analysis of differentially expressed genes between clone populations in patient 4. (**D**) The schematic diagram of the scClone strategy and its differences with the transcriptome expressional analysis workflow.

The results from the two spatial cases above demonstrated a degree of consistency between the genotype clusters inferred by scClone and the clusters of spatial spot transcriptomes (Figure 5C,I). However, unlike scRNA sequencing, the low utilization rate of ROI spots in spatial transcriptomics for scClone limited the resolution of clonal structures, as shown in a cutaneous squamous cell carcinoma sample (cSCC patient 6), where the sparse spots revealed two major clonal structures (Figure S9A–C). Consistent with the previous process, scClone identified two clonal clusters in another sample of cSCC (cSCC patient 4) (Figure S9D–F) [25]. Fortunately, scClone retained a high proportion of ROI spots here, and the sufficient spots allowed us to re-infer the clonal structure by utilizing transcriptomic annotations, ultimately obtaining nine subclonal clusters among seven transcriptomic groups (Figure 6A). Type_1_1 and type_1_2, which belonged to expressional cluster type_1, were identified as two distinct subclones by scClone based on their genotype differences. These two tumor subclones demonstrated consistency with their spatial positions, which cannot be distinguished by either gene expression or transcriptome-inferred CNVs (Figure S9G,H). Further enrichment and cell interaction analysis revealed that these two tumor subclones shared similar activities in the ACTIVIN and IL1 signaling pathways, while type_1_2 showed higher IL-10 and calcitriol activity with other spots (Figure 6B and Figure S9I). Type_1_1 exhibited high activity of pathways related to skin development and keratinocyte function, indicating a closer resemblance to normal skin cell functions. In contrast, type_1_2 was characterized by lipid metabolism, the NF-κB immune signaling pathway and immune cell activation (Figure 6C). Here we demonstrated that the integration of scClone and spatial transcriptomic information could provide a more in-depth understanding of the genetic and transcriptomic features of tumor subclones, thereby facilitating the diagnosis of complex clonal architectures and highlighting the clinical potential of scClone in tumor precision medicine.

## 3. Discussion

Single-cell mutational profiling emerges as a promising approach to studying clonal evolution compared with bulk VAF-based methods, and several lineage reconstruction algorithms have been developed, such as OncoNEM [38], SCITE [39], SiFit [40], and SiCloneFit [41]. These tools are developed for single-cell genome or exome sequencing data instead of scRNA-seq data, and they are mainly designed for scenarios with a small number of tumor cells. There are also some bioinformatic solutions for mutational inference from scRNA-seq, such as SCmut [42] and Cardelino [43], but they often require prior knowledge from matched bulk DNA sequencing data [43,44,45,46]. These strategies have not been widely used, as sampling bias between genomic and transcriptomic data may affect the reliability of clonal inference and assignment. More recently, some algorithms such as Monopogen [47] and SComatic [48] have been created for somatic mutation detection in scRNA-seq data, but they stop at variant detection: the sparse mutational landscapes they yield lack the statistical robustness for reliable reconstruction of clonal architecture and downstream evolutionary inference.

Our scClone is a completely de novo clonal structure inference toolkit for single-cell transcriptomics data, which is specially designed to avoid interference from RNA editing and allelic imbalance. It has a comprehensive set of functions, including mutation detection, clonal cluster identification, and clonal evolutionary analysis (Figure 6D). scClone could efficiently and simultaneously process thousands of single cells with different cell type identities, leveraging rich public scRNA-seq resources. Furthermore, it could be applied to spatial transcriptomics data with no need for prior knowledge from bulk or single-cell mutational profiling. scClone also incorporates the concept of mutational signatures from bulk sequencing and cell annotation from transcriptomics to intuitively present the clonal structure of a sample to users.

During the development of scClone, we have noticed that the number of raw mutations detected in scRNA-seq was often 4 to 5 orders of magnitude higher than mutations from bulk DNA sequencing, including a large number of sequencing errors and RNA-editing events. Even after strict quality control and filtering steps succeeded in preserving and revealing genuine mutations, the overlap between mutations identified by bulk sequencing and those derived from single-cell transcriptomes remained limited. This situation might arise from the fact that mutations occur constantly and randomly during cell division and most single-cell-specific mutations are too sparse to be detected in bulk sequencing with limited coverage. As evidence, the filtered mutations in our pipeline contain massive low-frequency mutations, which may be assigned to the neutral tail. Conversely, mutations detected by bulk genomic sequencing are rarely observed in single-cell transcriptomes due to various factors such as limited sequencing regions and coverage, differential expression and allelic imbalance. The reliable mutation detection from scRNA-seq remains a significant challenge today.

Designed to analyze mainstream single-cell and spatial transcriptomics datasets, the scClone workflow is compatible with prevailing single-cell transcriptomic data formats, features excellent extensibility, and can be directly applied to both mouse and human samples, aiming to ensure easy accessibility for various users. As a full-process computational tool for single-cell mutation detection, scClone consumes relatively substantial resources on personal servers; the resource consumption of all its processes in a cSCC sample (6000 cells) is provided in Figure S10A,B. Users can perform pre-screening to improve computational efficiency, such as filtering low-quality reads and excluding cells with low expression or unassigned cell types. Moreover, the loss of spatial resolution resulting from spot filtering remains an inherent limitation that the genome-based scClone framework is currently unable to resolve. One of the advantages is that spatial transcriptomics achieves higher mutation detection depth and higher mutation abundance within spots at the cost of cellular resolution (Figure S11A–D). Overall, our aim is to enable the exploitation of the genomic information hidden in the widespread scRNA-seq data, enabling researchers to link genetic and transcriptomic features and gain new insights into tumor initiation and progression.

## 4. Materials and Methods

### 4.1. Data Acquisition

No new sequencing data are generated in this study and all data are obtained from the following sources: The C1 single-cell transcriptome data and single-cell target sequencing data for hepatocellular carcinoma (HCC) were obtained from our previous work, easy to access with Gene Expression Omnibus (GEO) and Sequence Read Archive (SRA) under accession numbers GSE146115 and PRJNA606993 [8]. Myeloma data for the benchmark were obtained from GEO under accession numbers GSE106218 and GSE110499 [24]. The single-cell expression data of pancreatic cancer were acquired from GEO under accession GSE197177. Raw data of pancreatic cancer were obtained from Genome Sequence Archive under accession numbers HRA004556 (scRNA-seq) and HRA004625 (WES) [34]. The single-cell and spatial transcriptome data of cutaneous squamous cell carcinoma (cSCC) were acquired from GSE144240 [25]. The spatial transcriptome data of ovarian cancer were obtained from GSE211956 [37].

### 4.2. scClone Workflow

The first step of our tool is the detection and filtering of mutations (Figure 1B,C). We develop distinct pipelines for two major types of scRNA-seq raw data, full-length and 3′/5′ RNA sequencing, to obtain mutations per cell. These mutation sites are filtered using a support vector machine (SVM) to obtain high-confidence mutations. In this process, mutations cataloged in the dbSNP database with high prevalence are used as the positive dataset, while mutations recorded in the RNA editing database without aa-changed [26,49] and multi-base mutations serve as the negative dataset for training the identification of unknown mutations. The training features are derived from the VCF files of raw mutations.

The second step of scClone is inference of the genotype of each cell at each genomic site based on the mutations (Figure 1B,C). Given the low sequencing depth, the VAF values for the mutations will be transformed into positive real numbers of 0, 1, or 2 through beta-binomial distribution and allelic imbalance-based transformation. For the estimation of allelic imbalance, we consider expression rate and the database of human allelic expression [50]. Due to differential expression and limited capture counts for RNA transcripts, a sparse mutation-by-cell matrix is formed for each sample. We also employ a method of borrowing information from neighboring cells to fill in the drop-outs. As it is generally believed that genomic clonal identity should not contradict cell type, the parameters for filling in the drop-outs and identifying neighboring cells take the transcriptomic cell annotation and mutational signatures into account. This process forms a more detailed and informative mutation-by-cell matrix.

The third step aims to further denoise the matrix and uncover the clonal structure derived from single-cell transcriptomics (Figure 1B,C). Exclusive genotypes detected in a very few cells will be filtered out as noise through a robust principal component analysis (RPCA) process to make the clonal structure more observable. Finally, the accuracy and Gini score of all mutations for the initial hierarchical clusters are assessed using a random forest method, and mutations consistent with the clusters and cell annotations are used to draw the clonal structure diagram and reconstruct evolutionary trajectories (see Supplementary Materials for details).

## 5. Conclusions

- This work introduces scClone, a full-process clonal evolution inference toolkit based on mutation detection that relies solely on single-cell transcriptome sequencing. It includes a reliable mutation detection pipeline, a series of genotype inference algorithms, and clonal structure visualization.
- scClone achieves promising results across various cell types from different platforms and is compared with mainstream transcriptome analysis methods.
- scClone can be applied to spatial transcriptomics and identifies subclonal structures on histological sections that traditional methods fail to detect.

## Data Availability

This article does not generate any new sequencing data. The data presented in this study are available in NCBI GEO repository at https://www.ncbi.nlm.nih.gov/geo/ (accessed on 1 November 2023), reference numbers GSE146115, GSE106218, GSE110499, GSE197177, GSE144240 and GSE211956, for more details in Materials and methods. The source code in this article is available at https://github.com/monkeyBai96/scClone (accessed on 1 July 2025), with R package (version 1.0) in https://github.com/monkeyBai96/scClone/tree/main/package (accessed on 1 July 2025).

## Supplementary Materials

The following supporting information can be downloaded at https://www.mdpi.com/article/10.3390/ijms262311428/s1. References [26,28,32,49,51,52,53,54,55,56,57,58] are cited in the Supplementary Materials file.
