# Supplementary material for: De Novo Detection of Clonal Structure and Evolution in Single-Cell and Spatial Transcriptomes
Source: Int J Mol Sci. 2025 Nov 26;26(23):11428. doi: 10.3390/ijms262311428 (PMC12692173; doi:10.3390/ijms262311428)
Supplement: Supplementary file 1 [file ijms-26-11428-s001.zip › ijms-3980249-supplementary material-1.pdf]

International Journal of *Molecular Sciences*

MDPI

Supplementary materials

De Novo Detection of Clonal Structure and Evolution in Single-Cell and Spatial Transcriptomes

Shihao Bai †, Xianbin Su *,†, Ziyao Chen and Ze-Guang Han *

Key Laboratory of Systems Biomedicine (Ministry of Education), Shanghai Center for Systems Biomedicine, Shanghai Jiao Tong University, Shanghai 200240, China; b369259046@sjtu.edu.cn (S.B.); unicornczy@sjtu.edu.cn (Z.C.)
* Correspondence: xbsu@sjtu.edu.cn (X.S.); hanzg@sjtu.edu.cn (Z.-G.H.)
† These authors contributed equally to this work.

Academic Editor: Mauro Coluccia

Received: 27 October 2025
Revised: 20 November 2025
Accepted: 24 November 2025
Published: 26 November 2025

Citation: Bai, S.; Su, X.; Chen, Z.; Han, Z.-G. De Novo Detection of Clonal Structure and Evolution in Single-Cell and Spatial Transcriptomes. *Int. J. Mol. Sci.* **2025**, *26*, x. https://doi.org/10.3390/xxxxx

Copyright: © 2025 by the authors. Submitted for possible open access publication under the terms and conditions of the Creative Commons Attribution (CC BY) license (https://creativecommons.org/licenses/by/4.0/).

1. Supplementary methods

1.1. Classical analysis of single-cell and spatial transcriptomics

We initiate all our analysis from the raw data. The 10X Genomics single-cell transcriptome data are processed using CellRanger (v6.0.1), yielding both processed BAM files and a preliminary expression matrix. Similarly, the 10X Genomics spatial transcriptome data are processed using SpaceRanger (v3.1.1), resulting in BAM files, expression profiles and additional spatial images. CellRanger utilizes the GRCh37/hg19 reference genome, while SpaceRanger employs the GRCh38/hg38 reference genome as the original articles. Both genome references are packaged by 10X Genomics. For the full-length single-cell transcriptome sequencing of Fluidigm C1 platform, FASTQ files are generated for each single cell. After removal of rRNA reads, adaptors and low-quality reads, the sequencing reads are aligned to GRCh37/hg19 using TopHat (v2.0.13) and counted using Cufflinks (v2.2.1) [1]. The BAM files generated from the above processes are used for mutation detection and clonal inference with scClone, while the expression profiles are uniformly analyzed using Seurat (v5.1.0) [2]. After filtering, integration, dimension reduction and clustering, we reproduce the cell populations described in the original studies.

1.2. Gene markers and enrichment analysis

We extracted marker genes for each cluster from Seurat-derived expression-based clusters or scClone-derived mutation-based clusters using the FindAllMarker function in the Seurat package. Genes with p.adjust < 0.05 and |log2(fold change)| > 1 were defined as significant marker genes. Pathway enrichment analysis for Gene Ontology (GO) and Kyoto Encyclopedia of Genes and Genomes (KEGG) was performed using the ClusterProfiler R package (v4.8.3).

1.3. Insertion of artificial mutations

We utilized the addsnv.py and addindel.py scripts of the Bamsurgeon tool [3] to randomly introduce 1000 mutations into 400 myeloma cells, with an average of 2.5 mutations inserted per cell (comprising 900 single nucleotide variants [SNVs] and 100 insertions/deletions [INDELs]). Due to the inherent characteristics of single-cell RNA sequencing (scRNA-seq), the sequencing depth at each locus was typically 1~2×.

1.4. Cell-cell interaction analysis

CellChat (v2.1.2) [4] is employed to reveal the activity of specific pathways among cell clusters using its curated dataset of signaling pathway genes. The updated version of CellChat supports the analysis of spatial data, enabling the spatial visualization of interactions between spots on histological sections.

1.5. Copy number inference by single-cell expression data

We use all remained cells of a multiclonal cell type to infer copy number variations using inferCNV (v1.24.0) [5]. In the absence of standard normal cells for comparison, we use cells from the subclone with the fewest mutations as controls.

1.6. Pseudo-time analysis

Monocle2 (v2.28.0) [6] is utilized to construct a pseudo-time trajectory in selected cells or spots. Cells or spots are ranked based on differentially expressed genes along the inferred trajectory.

1.7. Mutational signature analysis

The 96 mutational signatures generated by single base substitutions (SBS) based on six base substitutions (C > A, C > G, C > T, T > A, T > C, and T > G) within 16 possible combinations of neighboring bases for each substitution are used as input to infer their mutational characteristic. We employ the sigminer R package (v2.3.1) [7] to calculate the signature of a series of mutations.

1.8. Statistical analysis and visualization

Statistical analyses are conducted using R software (v4.3.0). Unless otherwise specified, all statistical tests were performed using two-tailed Student's t-tests. Each group of data was derived from multiple independent replicate experiments. We utilize the ggplot2 (v3.5.1), ggsankey (v0.0.9), and pheatmap (v1.0.12) R packages to create figures.

2. scClone detailed workflow

2.1. Mutation calling and filtering

The alignment process with the reference genome is consistent with the classical transcriptome analysis. We perform quality filtering (MAPQ=60) on the processed BAM files generated by CellRanger and SpaceRanger and split the BAM files according to barcodes. For full-length sequencing, the default output is a BAM file for each individual cell. To save computational resources, we retain cells that are kept and annotated in the final transcriptome analysis. Cells with small BAM files (e.g., less than 2M) are also filtered out. For mutation calling, we select the sensitive Strelka (v2.9.2) tool [8], which provides more low-frequency mutations and quality scores. Annovar [9] is used for functional annotation of mutation sites.

We employ a support vector machine (SVM) to identify high-confidence mutations. Mutations overlapped with dbSNP and 1KG3 with high prevalence are used as the positive dataset, typically those found in more than 10 cells. We define multi-base mutations, consecutive SNVs (default >2) in genome, as the negative set. Moreover, mutations recorded in the RNA editing database without aa-changed [10, 11] are also considered as negative data. This is because multi-base mutations are rare events in the genome, and such occurrences in single cells are often due to sequencing errors. The training features are derived from the VCF files of mutation detection, including DP (filtered sequencing depth), QUAL (phred-scaled quality score), GQ (genotype quality), GQX (Empirically calibrated genotype quality score for variant sites), AD (allelic depths for the reference and alternative alleles), SB (sample site strand bias) and exonic functions.

SVM aims to find the optimal hyperplane that maximizes the margin between different classes in the feature space. Given a set of training data points $\{(x_i, y_i)\}_{i=1}^{n}$, where x_i is the feature vector and y_i is the class label, the goal of SVM is to find the hyperplane $w^T x + b = 0$ that maximizes the margin between the two classes. The optimization process can be formulated as:

$$\min_{w,b} \frac{1}{2} w^T w + C \sum_{i=1}^{n} \xi_i$$

$$\text{s.t.} \quad y_i (w^T x_i + b) \geq 1 - \xi_i$$

$$\xi_i \geq 0, \quad i = 1, 2, \ldots, n$$

where w is the weight vector, b is the bias term, C is the regularization parameter, and ξi are the slack variables. The decision function for classification is given by:

$$f(x) = \text{sign}(w^T x + b)$$

We used the linear kernel function for SVM, which is defined as:

$$K(x_i, x_j) = x_i^T x_j$$

The model derived from these training sets and features is used to predict the remaining mutation sites, which is trained by the svm function of e1071 R package. We compare the depth of mutation ALT and mutation signatures before and after SVM filtering.

2.2. Genotype inference

We filter mutation sites and cells based on richness and depth. For most datasets, we retain sites with mutations at a depth of > 30 in > 10 cells and cells with > 15 mutation sites. Retained mutations do not always imply high-depth mutations, as many low-depth mutations are preserved due to matching the requirements of sites. We keep these mutations because there are high confidence mutations at this locus that occur in other cells. According to the low depth, the conventional methods of calculating the variant allele frequency (VAF) is imprecise.

$$VAF = \frac{ALT}{ALT + REF}$$

where ALT is the number of alternate reads and REF was the number of reference reads.

We change the low-depth alternate depth and reference depth into a probability density ranging from 0 to 1 using beta-binomial distribution. That is, we determine the prior probability of the true genotype at that site based on the observed read counts.

$$Beta(p; \alpha, \beta) = \frac{p^{\alpha-1}(1-p)^{\beta-1}}{B(\alpha, \beta)} \quad \text{for} \quad 0 \leq p \leq 1$$

where p is the true VAF, same as p in Bernoulli trial, $\alpha - 1$ is the number of alternate reads and $\beta - 1$ is the number of reference reads. $B(\alpha, \beta)$ is a beta function.

VAFs, together with null values, are filled into a matrix $M = C \times V$ according to cells (C_i) and mutations (V_j). Subsequently, we infer genotype based on a transformer for VAF which considers allelic imbalance.

In this process, we construct a two-dimensional geometric space by defining a lower semicircle with a radius of 0.5 and center at (0.5, 0.5). The VAFs in the beta distribution are mapped as points on this semicircle, with the VAF value corresponding to the x-axis intercept. The transformed VAF (G_{ij}) is defined as the average probability density of the x-intercept, at which a line connecting the beta VAF point and the point (0.5, 0.5+θ_j). This transformation results in a broader range compared to the original beta VAFs, especially when the beta VAF approaches 0 or 1, where the transformed VAF can extend across the entire real number range. θ_j is a parameter that simulates the degree of allelic imbalance in transcriptional expression, with values ranging from 0 to 1. The higher degree of allelic imbalance constrains the range of transformed VAFs more significantly, finally reducing the likelihood of homozygous mutations. The allelic imbalance parameter is highly sensitive to the R_j and B_{ij}. Mutation sites with high expression rates in cells are less prone to occur allelic imbalance [12]. Due to the low-depth mutation detection, REF reads at most sites are often difficult to detect, leading to an apparent homozygous mutation. Therefore, B_{ij} typically represents all reference reads on the site. The detection of REF reads rapidly increases the allelic imbalance degree, indicating that the pure alteration reads at this site are more likely a result of the expression with allelic imbalance, thereby making the identification of homozygous mutations more stringent. For genes recorded in the database, we set the allelic imbalance to 0.5. Finally, considering the peak distribution of diverse datasets, we define the cutoffs for the transformed VAF at 0.2 and 1.2 for genotype rounding. (Genotype, 0: wild-type; 1: heterozygous mutation; 2: homozygous mutation).

$$G_{ij} = E(0.5 + \frac{(P_{ij} - 0.5) \cdot (\theta_j + 0.5)}{\theta_j + \sqrt{0.25 - (P_{ij} - 0.5)^2}})$$

$$\theta_j = \exp\left(-\frac{|A_j - B_j| \times R_j}{(B_j + 1)^3} \right)$$

$$A_j = \max(\sum_1^i ALT_{ij}, \sum_1^i REF_{ij})\ ;\quad B_j = \min(\sum_1^i ALT_{ij}, \sum_1^i REF_{ij})$$

$$f(G_{ij}) = \begin{cases} 0, & if\ \ G_{ij} < 0.2 \\ 1, & if\ \ 0.2 \le G_{ij} \le 1.2 \\ 2, & if\ \ G_{ij} > 1.2 \end{cases}$$

where G_{ij} is the genotype of cell C_i at mutation site V_j, P_{ij} is the distribution of beta-distribution VAF above, θ_j is the allelic imbalance at the mutation site V_j. A_j and B_j represent the maximum and minimum values of the total ALT reads and REF reads across all cells at site V_j, respectively. R_j is the rate of cells that have reads at the mutation site V_j. $f(G_{ij})$ refers to the inferred genotype.

To fill the numerous missing values, we next borrow information from neighboring cells and make the matrix smooth. For a given site V_j, the smoothed genotype is defined as:

$$G_{smoothed,ij} = (1-K_j)G_{inferred,ij} + K_j \times E(G_{neighbors,\,j})$$

$$K_j = k + (1-k)\mathbf{S}_j$$

$$\mathbf{S} = \frac{\mathbf{signature}}{N_{SNVs}}$$

K_j depends on the mutation signature formed by high-confidence mutations above. As INDELs do not contribute to the SBS signature, K_j is set to 0.1 by default. The value of k is a smoothness parameter that can be defined by users, with a general recommendation of 0.1. Neighbors are defined as cells with the same cell annotation (i.e., cell type) and a Manhattan distance of less than 20% of the mutation number from the target cell.

2.3. Clonal structure pruning

After the filtering and imputation steps, the retained cells and mutations exhibit an observable block structure in the "cell × mutation" matrix. This means the smoothed genotype-derived cell cluster can reflect the clonal structure. Before visualizing the clonal structure, noise removal is necessary to highlight the main structure of the clones. Robust Principal Component Analysis (RPCA) effectively filters sparse values in the matrix to obtain a modular matrix. RPCA is used to decompose a matrix into a low-rank component and a sparse component, which helps in filtering out noise and revealing the block structure.

$$\mathbf{X} = \mathbf{L} + \mathbf{S}$$

Low-rank component $\mathbf{L}$ reflects the main structure in the data, with a lower rank, and can be approximated by fewer singular values and corresponding singular vectors. Mathematically, the information is extracted through the singular value decomposition (SVD) of matrix $\mathbf{L}$:

$$L = U\Sigma V^{\top}$$

here, U and V are the matrices of left and right singular vectors, respectively, and Σ contains the singular values.

Sparse component $\mathbf{S}$ captures outliers or noise in the data, typically with many zero elements, hence "sparse". Mathematically, the non-zero elements in $\mathbf{S}$ are determined through soft thresholding:

$$S_{ij} = sign(X_{ij} - L_{ij}) \times \max\left(|X_{ij} - L_{ij}| - \lambda, 0\right)$$

here, λ is a threshold parameter that controls which elements are considered noise. By optimizing the following objective function, we obtain $\mathbf{L}$ and $\mathbf{S}$:

$$\min_{L,S} \|L\|_* + \lambda \|S\|_1 \quad \text{subject to } \mathbf{X} = \mathbf{L} + \mathbf{S}$$

$\|L\|_*$ is the nuclear norm of L. $\|S\|_1$ is the L_1 norm of L.

Due to the limitations of transcriptome data, the number of final mutations and cells is limited, making it unrealistic to obtain a precise clonal structure down to a few cells from the modular matrix. Here, we employ the random forest method, usually used as a classifier, to study the concordance of features (i.e., genotypes) with existing clones and cell types. This allows us to extract a matrix more supportive of the overall clonal structure,

thereby pruning the clonal structure. We use 70% of the cells as the training set, with cell type or current clonal cluster serving as the prediction targets. The random forest method constructs multiple decision trees and combines their results to improve predictive accuracy and control overfitting. Each tree is grown using a bootstrap sample of the training data, and for each node split, a subset of features is randomly selected to determine the best split. This introduces randomness and diversity among the trees, reducing the variance and increasing robustness. Hyperparameters include mtry (number of features considered per split) and ntree (number of trees in the forest), and their learning process are as follows:

mtry controls the number of features considered for splitting at each node. The optimal value is typically determined through five-fold cross-validation. A smaller mtry increases randomness, while a larger mtry reduces randomness but increases correlation among trees.

$$mtry = \sqrt{p}$$

ntree specifies the number of trees in the forest. Increasing ntree improves model stability and predictive performance. The optimal ntree is identified by monitoring the five-fold cross-validation error rate.

Feature importance is measured using Gini importance or permutation importance, helping to identify the most relevant features for classifying cell types and clonal clusters.

$$\text{Importance}(F) = \sum_{t=1}^{T} \sum_{s \in S(t)} \Delta L(s)$$

The refined matrix is constructed based on the Gini scores and accuracy of the features obtained from the random forest. Subsequently, the clonal structure is built within each cell type using the Manhattan distance. Clonal clusters with fewer than three cells are discarded.

References

1. Trapnell C, Roberts A, Goff L et al. Differential gene and transcript expression analysis of RNA-seq experiments with TopHat and Cufflinks, Nat Protoc 2012;7:562-578.
2. Satija R, Farrell JA, Gennert D et al. Spatial reconstruction of single-cell gene expression data, Nat Biotechnol 2015;33:495-502.
3. Ewing AD, Houlahan KE, Hu Y et al. Combining tumor genome simulation with crowdsourcing to benchmark somatic single-nucleotide-variant detection, Nat Methods 2015;12:623-630.
4. Jin S, Guerrero-Juarez CF, Zhang L et al. Inference and analysis of cell-cell communication using CellChat, Nat Commun 2021;12:1088.
5. Patel AP, Tirosh I, Trombetta JJ et al. Single-cell RNA-seq highlights intratumoral heterogeneity in primary glioblastoma, Science 2014;344:1396-1401.
6. Qiu X, Hill A, Packer J et al. Single-cell mRNA quantification and differential analysis with Census, Nat Methods 2017;14:309-315.
7. Tao Z, Wang S, Wu C et al. The repertoire of copy number alteration signatures in human cancer, Brief Bioinform 2023;24.
8. Kim S, Scheffler K, Halpern AL et al. Strelka2: fast and accurate calling of germline and somatic variants, Nat Methods 2018;15:591-594.
9. Wang K, Li M, Hakonarson H. ANNOVAR: functional annotation of genetic variants from high-throughput sequencing data, Nucleic Acids Res 2010;38:e164.
10. Kiran AM, O'Mahony JJ, Sanjeev K et al. Darned in 2013: inclusion of model organisms and linking with Wikipedia, Nucleic Acids Res 2013;41:D258-261.
11. Picardi E, D'Erchia AM, Lo Giudice C et al. REDIportal: a comprehensive database of A-to-I RNA editing events in humans, Nucleic Acids Res 2017;45:D750-D757.
12. Lu T, Park S, Zhu J et al. Overcoming Expressional Drop-outs in Lineage Reconstruction from Single-Cell RNA-Sequencing Data, Cell Rep 2021;34:108589.

Disclaimer/Publisher's Note: The statements, opinions and data contained in all publications are solely those of the individual author(s) and contributor(s) and not of MDPI and/or the editor(s). MDPI and/or the editor(s) disclaim responsibility for any injury to people or property resulting from any ideas, methods, instructions or products referred to in the content.
