# Supplementary material for: De Novo Detection of Clonal Structure and Evolution in Single-Cell and Spatial Transcriptomes
Source: Int J Mol Sci. 2025 Nov 26;26(23):11428. doi: 10.3390/ijms262311428 (PMC12692173; doi:10.3390/ijms262311428)
Supplement: Supplementary file 1 [file ijms-26-11428-s001.zip › ijms-3980249-supplementary material-2.pdf]

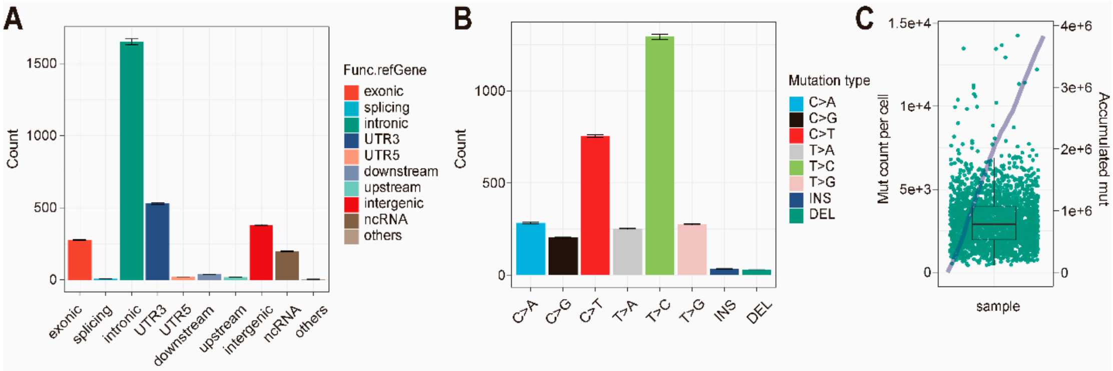

Figure S1. Mutation statistics in cSCC dataset. (A) Statistics of functional annotation for raw mutations detected by scClone in cSCC dataset (patient 6). Error bars represent standard errors. (B) Statistics of the nucleotide substitution types for mutations in cSCC dataset. Error bars represent standard errors. (C) Number of mutations in cSCC. Box and scatter plots are showing the number of mutations in each cSCC single cell (y-axis on the left), and the line is showing the cumulative number of mutations in all cSCC cells (y-axis on the right).

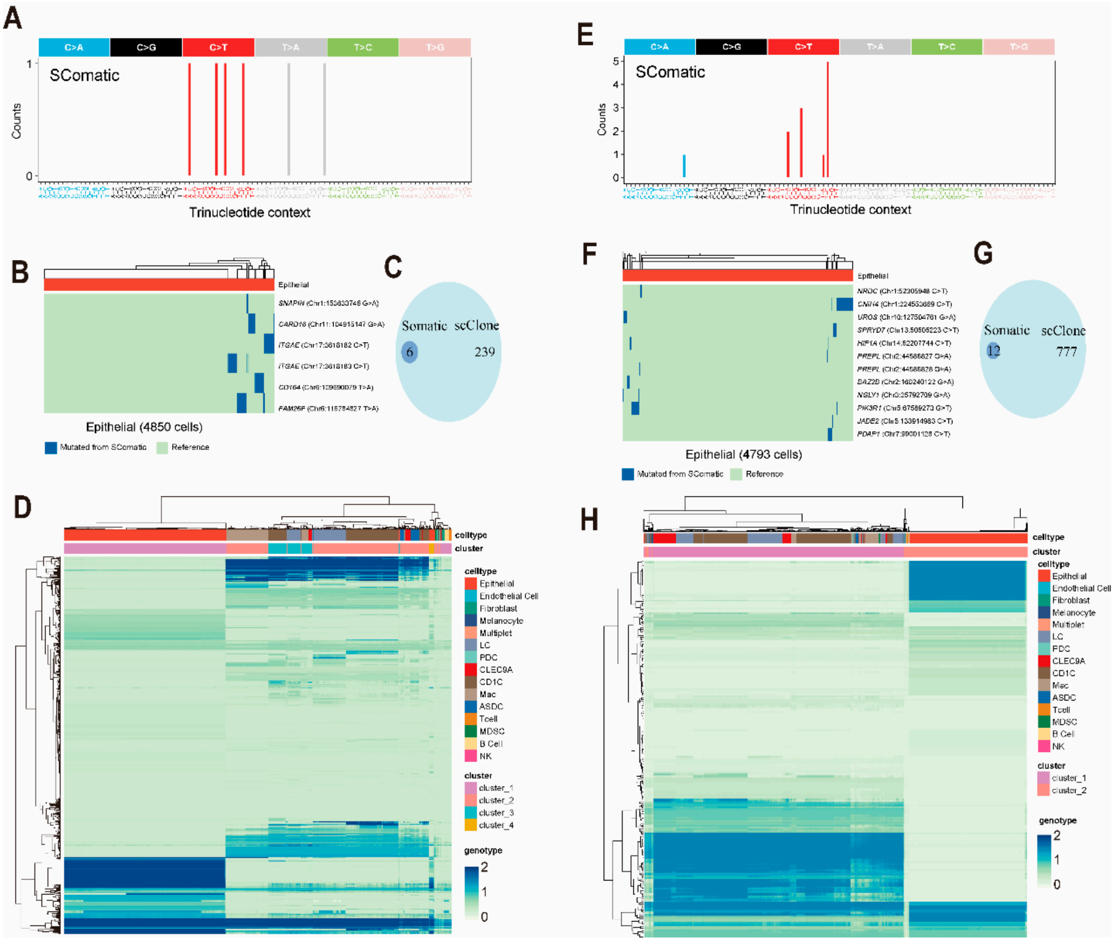

Figure S2. SComatic analysis of cSCC compared with scClone. (A&E) Mutational signatures from somatic mutations of SComatics in tumor samples of Patient 6 (A) and Patient 4 (E). (B&F) Cell-mutation heatmap with the somatic mutations from SComatics (B: Patient 6; F: Patient 4). (C&G) Venn plot of common mutations detected by SComatics and scClone (C: Patient 6; G: Patient 4). (D&H) Cell-mutation heatmap from scClone (D: Patient 6; H: Patient 4).

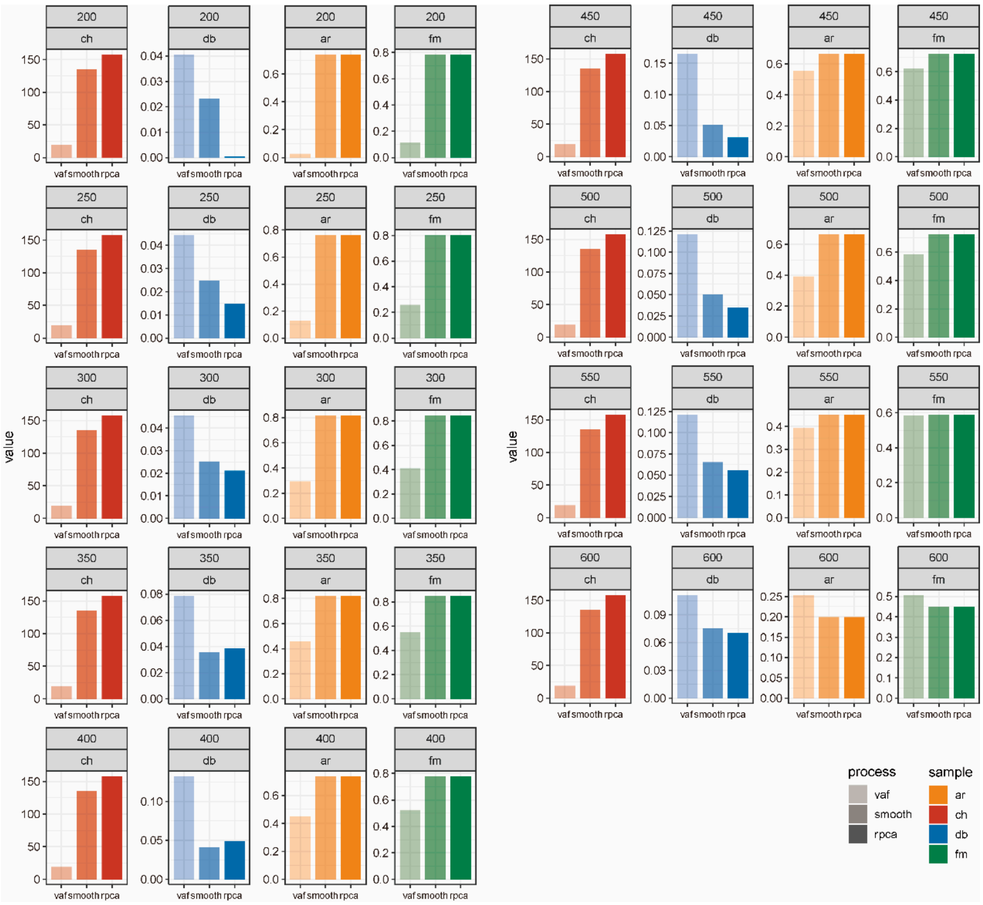

Figure S3. Sensitivity of scClone's clustering performance to different neighboring cell definition thresholds. AR: Adjusted Rand; CH: Calinski-Harabasz; DB: Davies-Bouldin; FM: Fowlkes-Mallows; NMI: Normalized Mutual Information.

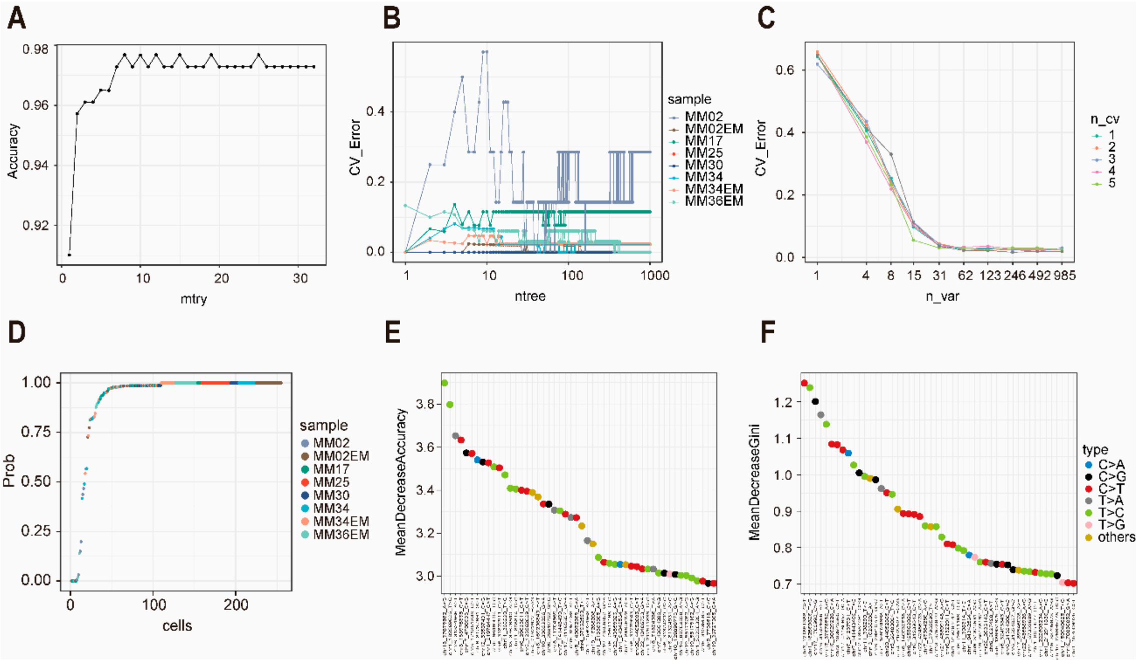

Figure S4. Random forest analysis of myeloma dataset. (A) Selection process for hyperparameter “mtry”. (B) Selection process for hyperparameter “ntree”. (C) Five-fold cross-validation to select the optimal number of features (variants here). (D) Probability of each cell being assigned to the right sample clusters. (E) Top 50 variant sites ranked by accuracy from highest to lowest. (F) Top 50 mutation sites ranked by Gini score from highest to lowest.

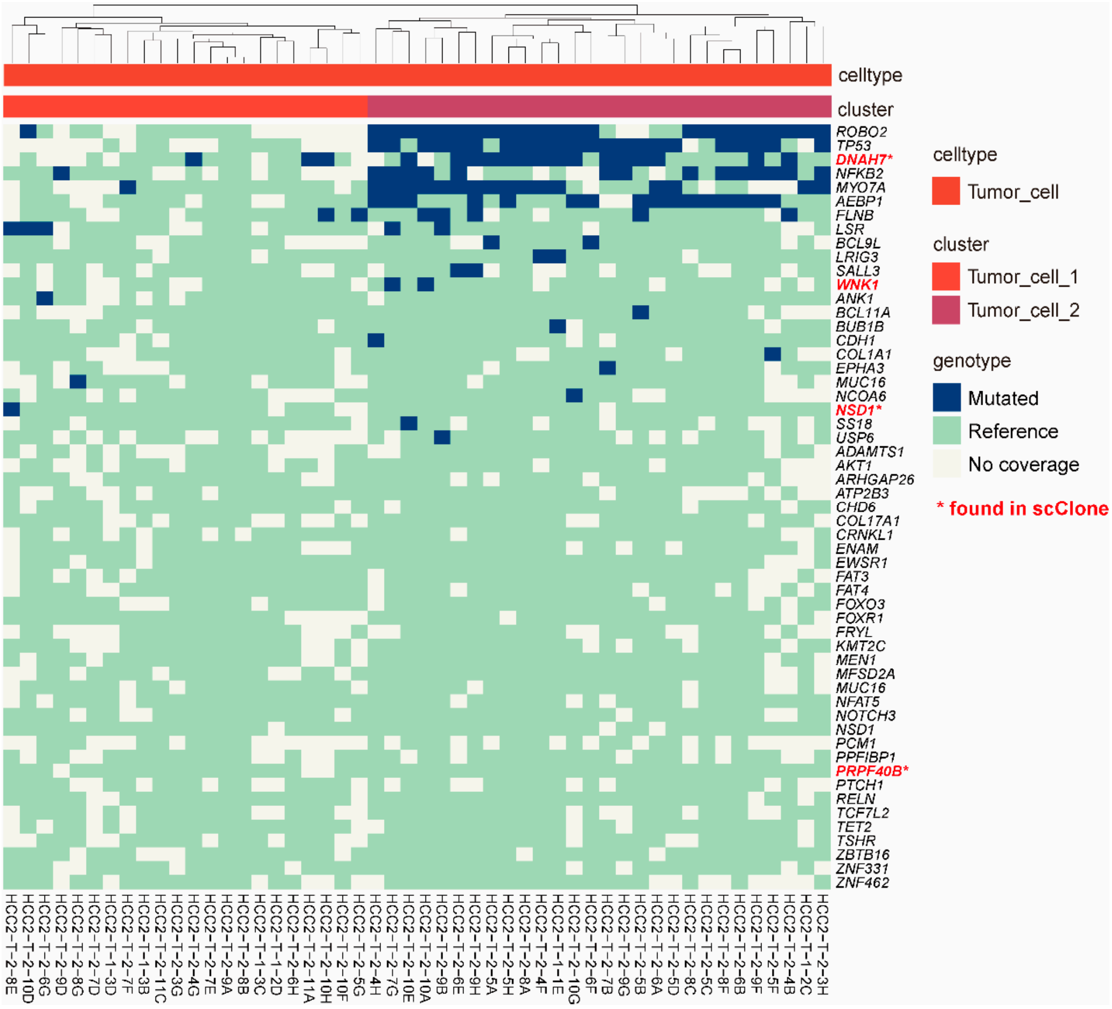

Figure S5. Cell-mutation heatmap from single-cell target sequencing. Cell-mutation heatmap with 50 cells and 55 mutation sites from scTarget-seq of Patient HCC2. Four mutation sites were found by scClone.

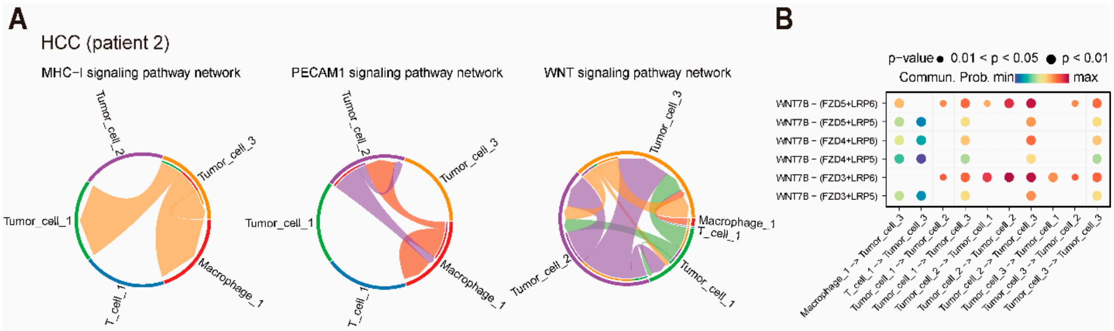

Figure S6. Interaction of signaling pathways among scClone-derived clones in HCC patient 2 (supplementary to Fig. 4). (A) Interactions between cell clones. (B) Expression of ligand-receptor pairs of WNT signaling pathway.

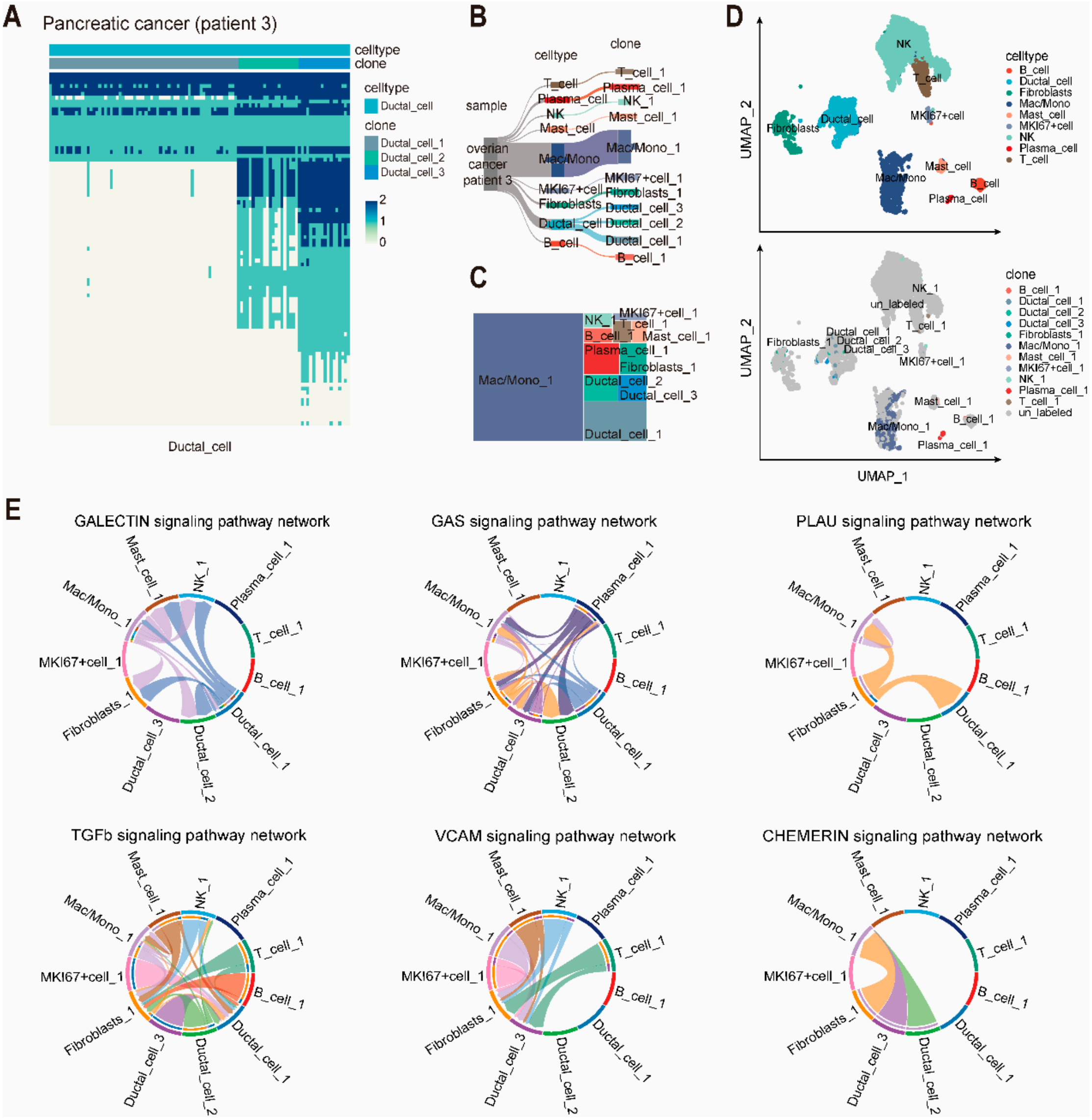

Figure S7. Application of scClone in single-cell transcriptomics in pancreatic cancer (supplementary to Fig. 4). (A) "Variant-cell" map of pancreatic cancer patient (patient 3). (B) Sankey diagram of cell types and clonal clusters in patient 3. (C) Intuitive visualization of clone sizes among all cells in patient 3. (D) UMAP of pancreatic cancer patient 3, with cell type (top) and clonal cluster information (bottom). (E) Interactions among cell clones in pancreatic cancer patient 3.

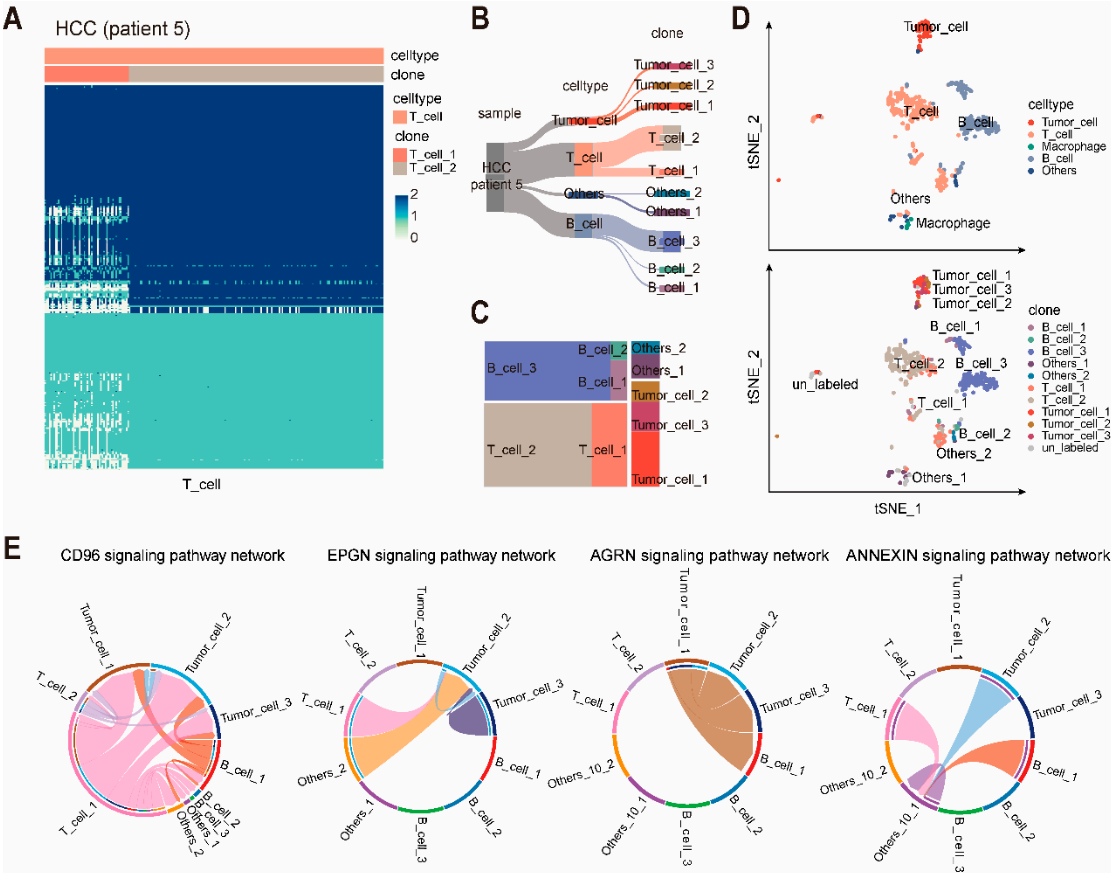

Figure S8. Application of scClone in single-cell transcriptomics in HCC patient 5 (supplementary to Fig. 4). (A) "Variant-cell" map of HCC patient (patient 5). (B) Sankey diagram of cell types and clonal clusters in patient 5. (G) Intuitive visualization of clone sizes among all cells in HCC patient 5. (D) tSNE map of HCC patient 5, with cell type (top) and clonal cluster information (bottom). (E) Interactions among cell clones in HCC patient 5.

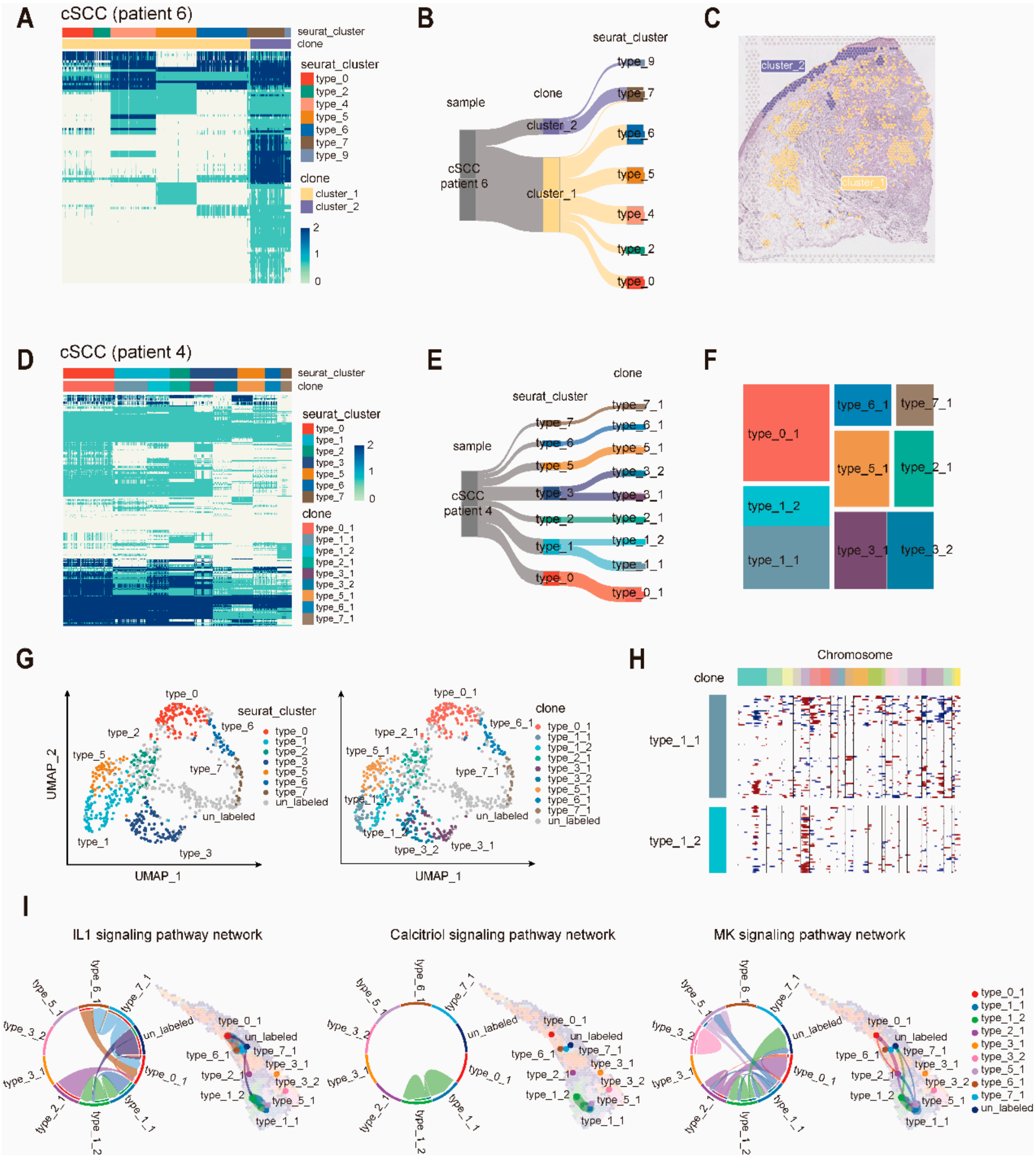

Figure S9. Application of scClone in spatial transcriptomics (supplementary to Fig. 6). (A) "Variant-cell" map of cSCC patient (patient 6). (B) Sankey diagram of cell types and clonal clusters in patient 6. (C) Clonal structure of spots displayed on pathological sections in patient 6. (D) " Variant-cell " map of cSCC patient (patient 4). (E) Sankey diagram of cell types and clonal clusters in patient 4. (F) Intuitive visualization of clone sizes among all cells in patient 4. (G) UMAP of patient 4, with clonal cluster (left) and transcriptional cluster information (right). (H) Whole-genome CNV inferred from single-cell transcriptome for patient 4, with each row representing a spot and each column representing a chromosomal bin. (I) Display of signaling pathway interactions between cells from patient 4 on pathological sections.

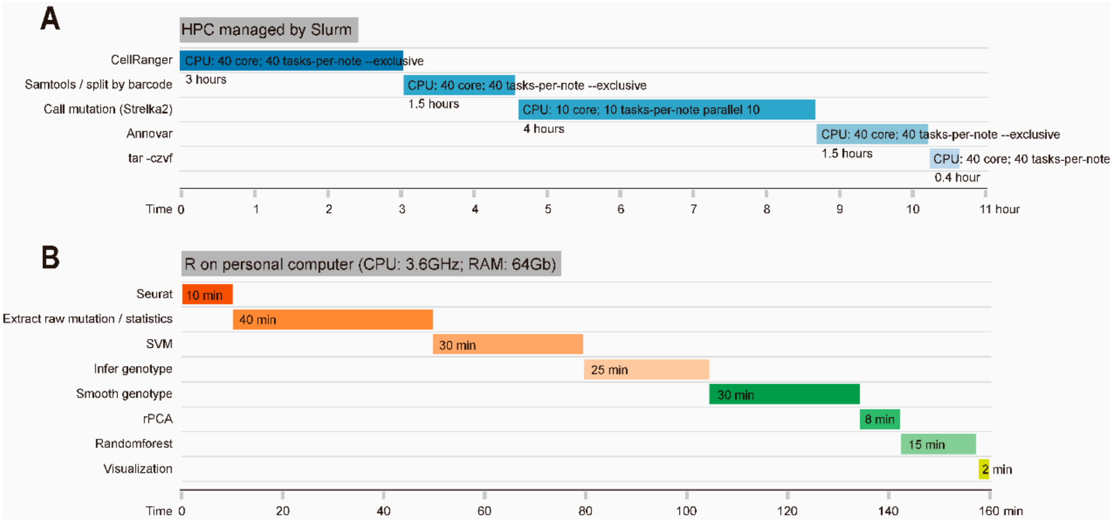

Figure S10. Computational resources consumed by the scClone workflow, using a cSCC sample with 6,000 cells as an example. (A) CPU and node requirements for each step on a Slurm-managed HPC (High Performance Computing). (B) Computational resources required in the R software on a personal computer.

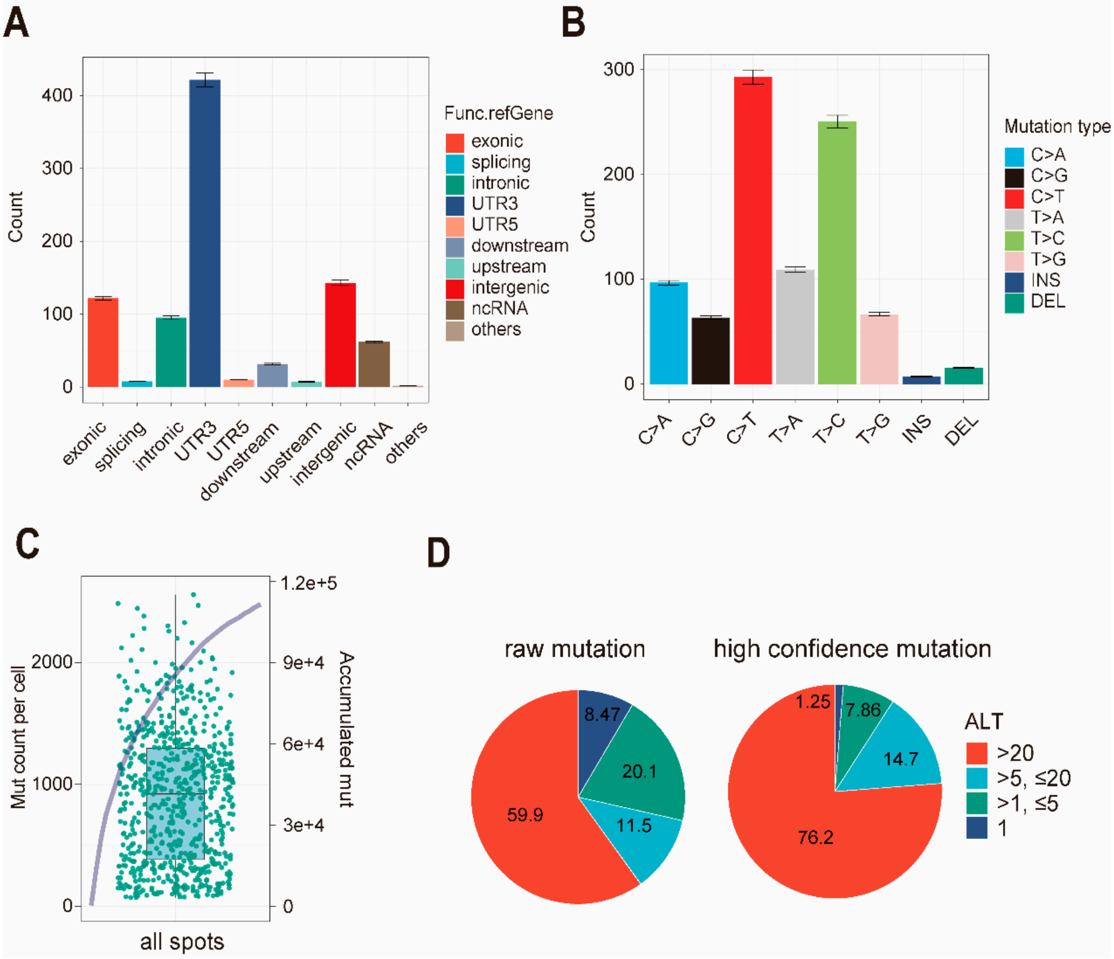

Figure S11. Mutation statistics in cSCC spatial transcriptome dataset. (A) Statistics of functional annotation for raw mutations in cSCC spots detected by scClone. Error bars represent standard errors. (B) Statistics of the nucleotide substitution types for raw mutations in cSCC spots. Error bars represent standard errors. (C) Number of mutations in cSCC spots. Box and scatter plots are showing the number of mutations in each cSCC spot (y-axis on the left), and the line is showing the cumulative number of mutations in all cSCC spots (y-axis on the right). (D) Fractions of mutations with ALT reads before and after SVM filtering in cSCC spatial transcriptome dataset, with raw mutations on the left and high-confidence mutations on the right.
